# Real‐world efficacy of treatment with benralizumab, dupilumab, mepolizumab and reslizumab for severe asthma: A systematic review and meta‐analysis

**DOI:** 10.1111/cea.14112

**Published:** 2022-03-09

**Authors:** David Charles, Jemma Shanley, Sasha‐Nicole Temple, Anna Rattu, Ekaterina Khaleva, Graham Roberts

**Affiliations:** ^1^ Academic Clinical Medicine Southampton General Hospital Southampton UK; ^2^ Child Health Southampton General Hospital Southampton UK; ^3^ Clinical Medicine Royal London Hospital London UK; ^4^ Clinical and Experimental Sciences and Human Development Faculty of Medicine University of Southampton Southampton UK; ^5^ Clinical and Experimental Sciences and Human Development University of Southampton Southampton UK

**Keywords:** asthma, asthma control, benralizumab, dupilumab, exacerbations, FeNO, FEV_1_, mepolizumab, real‐world studies, reslizumab

## Abstract

**Background:**

Severe asthma is a major cause of morbidity. Some patients may benefit from biological therapies. Most evaluations of these treatments are derived from randomized controlled trials (RCTs), but few patients are eligible for these trials. Studies involving more diverse groups of participants exist, but there is a lack of precise pooled estimates.

**Objective:**

This systematic review aims to evaluate the real‐world efficacy of recently and nearly licensed biological therapies for severe asthma to assess the generalizability of the RCT data.

**Methods:**

Clinical outcomes including exacerbation rate, oral corticosteroid usage, forced expiratory volume in 1 second (FEV_1_) and fractional exhaled nitric oxide (FeNO) were examined. Studies were assessed for risk of bias using the Critical Appraisal Skills Programme checklist tool. The certainty of evidence was assessed using Grading of Recommendations, Assessment, Development and Evaluations (GRADE).

**Results:**

A total of 21 studies examining biologicals in real‐world settings were identified; they mostly focused on benralizumab and mepolizumab. The introduction of biologicals reduced the annualized exacerbation rate significantly by −3.79 (95% confidence interval [CI] −4.53, −3.04), −3.17 (95% CI −3.74, −2.59) and −6.72 (95% CI −8.47, −4.97) with benralizumab, mepolizumab and reslizumab, respectively. Likewise, improvements were observed in FEV_1_ (0.17 L 95% CI 0.11, 0.24) and FeNO (−14.23 ppb 95% CI −19.71, −8.75) following the treatment with mepolizumab. After treatment with benralizumab, there was an increase in FEV_1_ (0.21 L 95% CI 0.08, 0.34).

**Conclusions:**

These data demonstrate that anti‐IL5 biologicals may improve the clinical outcomes of patients with severe asthma in a clinic environment with similar effect sizes to RCTs. The data were mainly retrospective and unadjusted, so estimated effect sizes may not be reliable. More data are needed to acquire accurate effect estimates in different subpopulations of patients.

## Graphical abstract

This study examines the evidence for the efficacy of biologics in typical patients with severe asthma. Many of these patients would not have qualified for randomized controlled trials (RCTs). We demonstrate that biologicals are effective for typical patients and that across certain outcomes including forced expiratory volume in 1 second, the gains are comparable to results from RCTs.

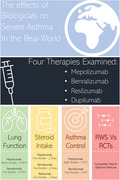

**Key messages**

- Observational data from typical populations can help to establish the generalizability of results observed in randomized controlled trials (RCTs).
- Treatment with biologicals is associated with significant improvements in asthma control, exacerbations and lung function.
- The improvement in results is comparable to RCTs, implying RCTs are applicable to typical populations.

## 1 INTRODUCTION

Asthma is a heterogenous non‐communicable chronic disease which is characterized by wheeze, shortness of breath, cough and chest tightness. Approximately, 5%–10% of patients have severe asthma, a form associated with increased mortality, reduced quality of life and increased healthcare costs.^1^, ^2^ According to the American Thoracic Society/European Respiratory Society (ATS/ERS) guidelines, asthma can be defined as severe when it requires high doses of inhaled corticosteroids and a second controller agent (which can include oral corticosteroid [OCS]) to prevent it from becoming uncontrolled, or when it is uncontrolled despite this therapy.^3^ In patients with asthma, clinical objectives include reducing the rate of exacerbation, improving symptom control and reducing the use of oral corticosteroids.^4^ Recent developments in our understanding of the molecular biology of asthma has facilitated new treatment options.

Asthma can be subdivided into endotypes based on the pathophysiology observed in patient populations. In some groups, activated Type 2 T Helper cells produce Interleukin‐4 (IL‐4), Interleukin‐5 (IL‐5) and Interleukin‐13 (IL‐13) which act as the principal drivers of inflammation.^5^ ILC2 cells are another important source of these inflammatory cytokines. These cells are activated by alarmins produced by epithelial cells in response to various biological molecules.^6^ IL‐5 is involved in the maturation of eosinophils and in the migration of eosinophils to the lungs, where they trigger inflammation and cause hyper‐responsiveness of the airways.^7^ IL‐4 and IL‐13 interact with the IL‐4Ra receptor subchain and stimulate the production of immunoglobulin E and mediators of airway remodelling.^8^ IL‐13 also modulates nitric oxide production within the respiratory system, increases mucus production and increases smooth muscle contractility.^8^ Monoclonal antibodies target these pathways, mepolizumab and reslizumab bind to IL‐5, benralizumab interacts with the IL‐5 receptor and dupilumab binds to the shared component of the IL‐4/IL‐13 receptor.^9^, ^10^ These biological therapies disrupt the action and activities of these key molecules.

Biologicals have been shown to be effective in randomized controlled trials (RCTs). However, it has been recognized that only a minority of patients with severe asthma would also be eligible for inclusion within an RCT.^11^ Moreover, in RCTs, many patients randomized to the placebo arm experience a significant improvement in asthma control and a reduction in the number of exacerbations suggesting that these apparent severe asthmatics simply require increased monitoring rather than increase drug therapy.^12^ Consequently, there are questions over the generalizability of results from RCTs. It has been suggested that, in general, the conclusions derived from RCTs could be more impactful if supported by evidence of therapeutic effectiveness from clinical practice. As such, real‐world studies are becoming more influential, informing decisions by healthcare regulatory bodies including the National Institute of Clinical Excellence (NICE) and Germany's Institute for Quality and Efficiency in Health Care.^13^, ^14^ However, when assessing real‐world studies, their small sample size and, in some cases, the lack of a control group can limit interpretation. These can both be addressed within the context of a systematic review and meta‐analysis through reference to effect measures derived from RCTs and by collating and pooling multiple studies to provide precise effect estimates. In this systematic review, we aimed to evaluate the efficacy of recently licenced biological agents in populations of participants with severe asthma reported in real‐world studies to assess the generalizability of the RCT data.

## 2 METHODS

### 2.1 Protocol and registration

The study protocol was registered on the International Prospective Register of Systematic Reviews (PROSPERO, CRD42020207080). Grading of Recommendations, Assessment, Development and Evaluations (GRADE) and Preferred Reporting Items for Systematic Reviews and Meta‐Analyses guidelines were followed.^15^, ^16^ The review aimed to assess adults and children with a diagnosis of asthma from 6 years of age treated with any nearly licenced or licenced biological therapies. Clinical outcomes examined included exacerbation rate, asthma control and reduction in the mean daily dose of OCS. Additional outcomes examined included forced expiratory volume in one second (FEV_1_), fractional exhaled nitric oxide (FeNO) and eosinophil count. Benralizumab, mepolizumab, reslizumab and dupilumab were examined. Omalizumab was excluded from this analysis as it has been in routine use for over a decade, and the authors chose to focus on newer agents where healthcare professionals have less clinical experience.

### 2.2 Search strategy and study selection

Medline (OVID), Embase (OVID), the Cumulative Index to Nursing and Allied Health Literature (CINAHL) database (EBSCOhost) and ISI Web of Science (WOS) were systematically searched to identify eligible real‐world trials examining biologicals in asthma. The search strategy combined relevant Medical Subject Headings (MeSH) and plain text. The search strategies (Supporting Information S1) were adapted for each of the databases. Databases were searched from inception to 31 August 2020. Searches were restricted to articles published in English. Two independent researchers (D. C. and J. S.) then selected relevant records by a two‐step process. Initially, titles and abstracts of records were screened for eligibility. The full text of any eligible record was then acquired, and a detailed evaluation was performed. Any disagreements about inclusion were resolved by discussion and consensus, or by consultation with a third reviewer (G. R.). Studies were excluded if they were very small (included <20 participants in total) to minimize bias, were animal studies, qualitative research, systematic reviews, narrative reviews, RCTs, editorials, conference abstracts, studies not relating to asthma, studies not relating to specified biologics, studies not in English or studies not focused on real‐life research. References were managed with Endnote Version X9 (Thomson Reuters) and Rayyan.^17^

### 2.3 Data extraction and risk of bias assessment

Two reviewers (D. C. and S. N. T.) independently extracted the characteristics and data from the eligible studies. These data sets were checked against each other, and any disagreements were resolved by arbitration and consensus or by consultation with a third reviewer (G. R.). Where necessary authors were contacted and requested to provide additional data, including unadjusted results. Risk of bias of included studies was evaluated with the use of the Critical Appraisal Skills Program tool for cohort studies.^18^ Disagreements were solved through discussion; if agreement could not be reached, arbitration with a third reviewer (G. R.) was held.

### 2.4 Meta‐analysis

Descriptive tables that included information on population characteristics, interventions and key outcomes were created. Data were pooled and meta‐analyzed using STATA^19^ using a random effects model. As outcomes were continuous, mean differences (MD) with 95% confidence intervals (CI) were used. Where insufficient data were present to perform a meta‐analysis, authors were contacted by members of the review team to request additional data. Heterogeneity was calculated by the *I* ^2^ statistic. Heterogeneity around baseline eosinophilia was explored by meta‐regression owing to the possibility of variation in real patient populations.

### 2.5 GRADE assessment

This study assessed the strength of outcome as per the GRADE guidelines.^16^ As this study dealt with observational studies, all studies were initially given two of four points. This was adjusted by deducting points if there was risk of bias, inconsistency, publication bias, imprecision or indirectness. Points were added as per the quality assessment criteria, for example if large effects were seen or if there was evidence of a dose response. Based on these, we assigned outcomes to four categories of certainty based on the overall GRADE score for each comparison. Outcomes were categorized as high (at least 4 points), moderate (3 points), low (2 points) and very low certainty (1 point or less).

### 2.6 Comparison to RCTs

Studies examined by a recent systematic review^20^ were selected and data on the effect size for key measures: FEV_1_, exacerbation and control, amongst the active treatment group were extracted. Where required authors were contacted, and data were obtained. In cases where this was not possible data points were estimated from figures using Graph Grabber 2.0 (Quintessa).

## 3 RESULTS

### 3.1 Search results

**FIGURE 1:**
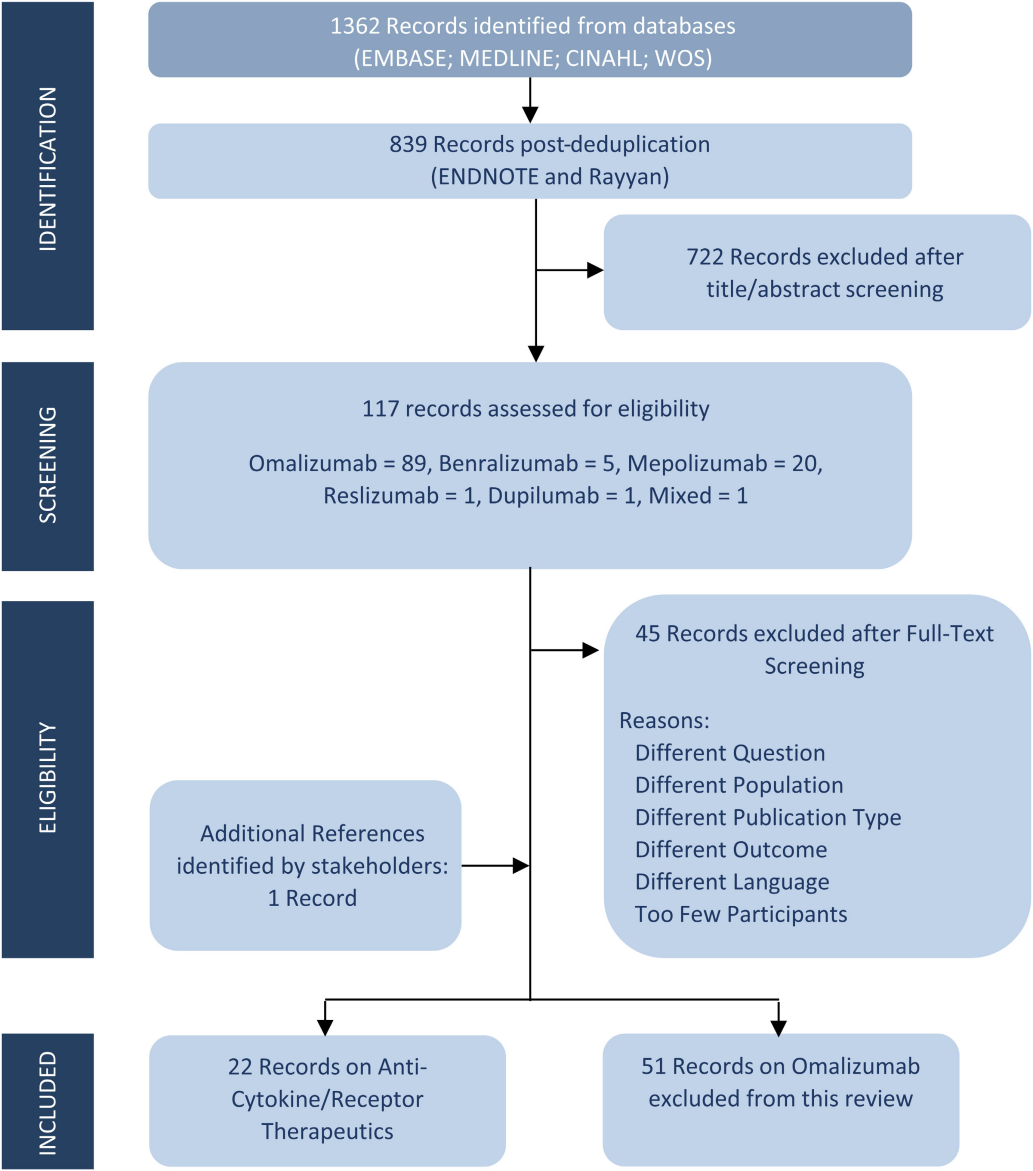
Prisma flow diagram. Study flow chart illustrating the selection of evidence. Records on omalizumab excluded from this review as it has been licensed for over a decade

The search strategy retrieved 1392 records. After duplicates were excluded, 839 unique records were identified. A total of 117 records were appraized in full. A total of 51 records were found to relate to omalizumab and so were considered beyond the scope of this review. Additionally, 45 further records were excluded due to differences in the population, study designs, outcome, dosage or route, inclusion of too few participants (<20 total participants), publication type or language. A total of 22 studies met the inclusion criteria. Of these, 5 investigated benralizumab,^21^, ^22^, ^23^, ^24^, ^25^ 14 discussed mepolizumab,^26^, ^27^, ^28^, ^29^, ^30^, ^31^, ^32^, ^33^, ^34^, ^35^, ^36^, ^37^, ^38^ 1 focused on reslizumab^39^ and 1 paper examined dupilumab^40^ (Figure 1). One study examined three biologicals; mepolizumab, benralizumab and reslizumab.^41^

### 3.2 Characteristics of included studies

The main characteristics of the studies are included in Tables [Link], [Link], [Link], [Link]. The real‐world trials included 1512 participants who were followed for up to 2 years. All studies recruited adults. Mean ages for participants ranged between 52 and 60 years. All studies were conducted between 2018 and 2020. The percentage of women in the studies ranged between 78.6% and 34%. Of the studies that reported the number of smokers, the percentage ranged between 0% and 41%. Most studies were retrospective studies apart from three studies examining mepolizumab (Table S16). Approximately, 47.6% of the studies use the ERS/ATS definition of severe asthma, 9.5% of studies used the Global Initiative of Asthma guideline definition whilst in 42.9% of studies, severe asthma was physician diagnosed.

### 3.3 Risk of bias of included studies and publication bias

Four studies were found to have a low risk of bias.^24^, ^31^, ^35^, ^38^ One study was found to have a high risk of bias.^36^ The remaining studies were deemed to be at moderate risk of bias (Tables [Link], [Link], [Link]). Studies were grouped according to biological agent and clinical outcome and assessed using funnel plots (Figures [Link], [Link], [Link], [Link], [Link]). Publication bias was incorporated into the GRADE assessment (Tables [Link], [Link], [Link]).

### 3.4 Change in asthma exacerbation

Data pertaining to asthma exacerbation rate were found for all four biological therapies. Seven studies reported asthma exacerbation data in people treated with mepolizumab.^30^, ^31^, ^34^, ^35^, ^37^, ^38^, ^41^ Three studies examined asthma exacerbations in patients treated with benralizumab.^24^, ^25^, ^41^ Two studies examined asthma exacerbations in people treated with reslizumab.^39^, ^41^ In these 12 studies, biologicals reduced annualized exacerbation rate with moderate certainty as assessed using the GRADE criteria. When compared to baseline, annualized exacerbation rate was reduced with mepolizumab, benralizumab and reslizumab by −3.17 [95% CI −3.74; −2.59], −3.79 [95% CI −4.53; −3.04] and −6.72 [95% CI −8.47; −4.97], respectively. Significant heterogeneity was noted for mepolizumab, meta‐regression demonstrated a potentially weak association between baseline eosinophilia and effect size (*p* = .063) (Tables 1, 2, 3; Table S9).

**TABLE 1:** Summary of results for mepolizumab

Outcome	No. of participants/number of trials evaluated for an outcome	Certainty of the evidence (GRADE)	Pooled effect (95% CI)
ΔAnnualized rate of asthma exacerbation	7 Trials^30^, ^31^, ^34^, ^35^, ^37^, ^38^, ^41^ 458 Participants	Moderate	−3.17 [−3.74, −2.59]
ΔACT score	8 Trials^27^, ^28^, ^30^, ^32^, ^33^, ^34^, ^35^, ^41^ 533 Participants	Low	6.15 [5.14, 7.15]
ΔACQ‐6 score	2 Trials^31^, ^38^ 157 Participants	Very low	−0.53 [−0.76, −0.30]
ΔOral steroid dose (mg)	5 Trials^30^, ^31^, ^32^, ^33^, ^34^, ^41^ 325 Participants	Very low	−5.30 [−7.50, −3.10]
ΔFEV_1_ (L)	7 Trials^27^, ^28^, ^31^, ^32^, ^33^, ^34^, ^41^ 341 Participants	Low	0.17 [0.11, 0.24]
ΔFeNO (ppb)	7 Trials^27^, ^28^, ^32^, ^33^, ^34^, ^35^, ^38^ 363 Participants	Moderate	−14.23 [−19.71, −8.75]
ΔBlood eosinophils (cells/µl)	7 Trials^27^, ^28^, ^31^, ^32^, ^33^, ^34^, ^35^, ^41^ 466 Participants	Very low	−609.19 [−793.20, −425.18]

Note

Evidence examined using the GRADE Criteria where High Confidence indicates that the true effect lies close to that of the estimate of the effect, Moderate Confidence indicates the true effect is likely to lie close to the estimate of the effect, but there is a possibility that it is substantially different, Low Confidence indicates the true effect may be substantially different from the estimate of the effect, Very Low Confidence indicates the true effect is likely to be substantially different from the estimate of effect.

The minimal clinically important difference (MCID) for FEV_1_ is 0.2 L as per previous studies.^19^ The MCID for ACT is 3 as per previous studies.^42^ Pooled effects represent mean difference [95% confidence interval] from a random effects meta‐analysis model after therapy with mepolizumab in real‐world studies.

Abbreviations: ACT, Asthma Control Test (higher scores indicating better control); CI, confidence interval; FeNO, fractional exhaled nitric oxide; FEV1, forced expiratory volume in 1 s.

**TABLE 2:** Summary of results for reslizumab

Outcome	No. of participants/number of trials evaluated for an outcome	Certainty of the evidence (GRADE)	Pooled effect (95% CI)
ΔAnnualized rate of asthma exacerbation	2 Trials^39^, ^41^ 24 Participants	Moderate	−6.72 [−8.47, −4.97]
ΔOral steroid dose (mg)	2 Trials^39^, ^41^ 24 Participants	Low	−3.90 [−5.26, −2.54]
ΔBlood eosinophils (cells/µl)	2 Trials^39^, ^41^ 24 Participants	Very low	−603.60 [−838.69, −368.51]

Note

Evidence examined using the GRADE Criteria where High Confidence indicates that the true effect lies close to that of the estimate of the effect, Moderate Confidence indicates the true effect is likely to lie close to the estimate of the effect, but there is a possibility that it is substantially different, Low Confidence indicates the true effect may be substantially different from the estimate of the effect, Very Low Confidence indicates the true effect is likely to be substantially different from the estimate of effect.

Pooled effects represent mean difference [95% confidence interval] from a random effects meta‐analysis model after therapy with Reslizumab in real‐world studies.

Abbreviations: CI, confidence interval.

**TABLE 3:** Summary of results for benralizumab

**Outcome**	**No. of participants/number of trials evaluated for an outcome**	**Certainty of the evidence (GRADE)**	**Pooled effect (95% CI)**
ΔAnnualized rate of asthma exacerbation	3 Trials^24^, ^25^, ^41^ 157 Participants	Moderate	−3.79 [−4.53, −3.04]
ΔACT score	4 Trials^22^, ^23^, ^25^, ^41^ 93 Participants	Very low	5.82 [3.39, 8.25]
ΔOral steroid dose (mg)	5 Trials^23^, ^24^, ^25^, ^31^, ^41^ 107 Participants	Very low	−8.35 mg [−13.83, −2.87]
ΔFEV_1_ (L)	5 Trials^22^, ^23^, ^24^, ^25^, ^41^ 207 Participants	Low	0.21 L [0.08, 0.34]
ΔFeNO (ppb)	3 Trials^22^, ^23^, ^24^ 179 Participants	Low	−14.18 [−36.54, 8.17]
ΔBlood eosinophils (cells/µl)	5 Trials^22^, ^23^, ^24^, ^25^, ^41^ 215 Participants	Very Low	−518.68 [−820.24, −217.12]

Note

Evidence examined using the GRADE Criteria where High Confidence indicates that the true effect lies close to that of the estimate of the effect, Moderate Confidence indicates the true effect is likely to lie close to the estimate of the effect, but there is a possibility that it is substantially different, Low Confidence indicates the true effect may be substantially different from the estimate of the effect, Very Low Confidence indicates the true effect is likely to be substantially different from the estimate of effect.

The minimal clinically important difference (MCID) for FEV_1_ is 0.2 L as per previous studies.^19^ The MCID for ACT is 3 as per previous studies.^42^ Pooled effects represent mean difference [95% confidence interval] from a random effects meta‐analysis model after therapy with benralizumab in real‐world studies.

Abbreviations: ACT, asthma control test (higher scores indicating better control); CI, confidence interval; FEV_1_, forced expiratory volume in one second; FeNO, fractional exhaled nitric oxide.

### 3.5 Lung function

FEV_1_ change was assessed following the treatment with mepolizumab and benralizumab. There was low certainty of evidence of an increase in FEV1 after treatment with these two agents. Seven studies examined the change in FEV_1_ after treatment with mepolizumab; an increase of 0.17 L [95% CI 0.11; 0.24] was observed.^27^, ^28^, ^31^, ^32^, ^33^, ^34^, ^41^ Five studies reported the change in FEV_1_ after benralizumab treatment^22^, ^23^, ^24^, ^25^, ^41^; a 0.21 L [95% CI 0.08; 0.34] increase was seen. Heterogeneity in effect size for participants given benralizumab was explored and found to be associated with baseline eosinophilia (*p* = .003) (Tables 1, 2, 3; Table S9).

### 3.6 Asthma control

Asthma control was evaluated in studies using the Asthma Control Test (ACT) and the Asthma Control Questionnaire (ACQ‐6). An improvement was seen in asthma control with mepolizumab and benralizumab, with low and very low certainty of evidence, respectively. Eight studies assessed the effect of mepolizumab on ACT.^27^, ^28^, ^30^, ^32^, ^33^, ^34^, ^35^, ^41^ These studies demonstrated a 6.15 point [95% CI 5.14, 7.15] improvement. Two studies examined the effect of mepolizumab on ACQ‐6.^31^, ^38^ A −0.53 point [−0.76; −0.30] improvement was observed. Four trials examined the effect of benralizumab on ACT.^22^, ^23^, ^25^, ^41^ A 5.82 point [3.39, 8.25] improvement was seen, which was noted to be greater than the minimal clinically important difference.^42^ Limited data for the effect of benralizumab on ACQ‐6 were acquired, and one study demonstrated a −0.78 [−1.02, −0.54] improvement in ACQ‐6 score.^24^ Significant heterogeneity was seen in the effect sizes for studies where participants were treated with both mepolizumab and benralizumab. The heterogeneity was explored and found to be unrelated to baseline eosinophilia in the case of mepolizumab (*p* = .544) but related in the case of benralizumab (*p* < .001) (Tables 1, 2, 3; Table S9).

### 3.7 Changes in oral steroids

Studies analyzed the change in mean daily steroid doses following the treatment with mepolizumab, benralizumab and reslizumab. Although a decrease was found with all three agents, the quality of evidence was found to be very low for mepolizumab and benralizumab and low for reslizumab. Five trials examined treatment with mepolizumab,^30^, ^31^, ^32^, ^33^, ^34^, ^41^ five trials studied treatment with benralizumab^23^, ^24^, ^25^, ^31^, ^41^ and two trials explored treatment with reslizumab.^39^, ^41^ Those who were taking mepolizumab were found to have a reduction in mean daily OCS dose (−5.30 mg [95% CI −7.50; −3.10]). A similar reduction was found for both benralizumab and reslizumab (−8.35 mg [95% CI −13.83; −2.87]; −3.90 mg [95% CI −5.26; −2.54]). Analysis of both benralizumab and mepolizumab demonstrated significant heterogeneity, although less heterogeneity was observed in studies assessing reslizumab. Baseline eosinophilia did not explain the heterogeneity seen in the analysis of studies assessing mepolizumab (*p* = .909) or benralizumab (*p* = .129) (Tables 1, 2, 3; Table S9).

### 3.8 Change in eosinophils

Blood eosinophilia was assessed after treatment with three biologicals. Each demonstrated a decrease, but the quality of evidence was found to be very low in the case of mepolizumab, benralizumab and reslizumab. Eight trials examined mepolizumab,^27^, ^28^, ^31^, ^32^, ^33^, ^34^, ^35^, ^41^ five trials examined benralizumab^22^, ^23^, ^24^, ^25^, ^41^ and two trials examined reslizumab.^39^, ^41^ The use of mepolizumab, benralizumab and reslizumab was associated with a reduction in eosinophils (−609.19 cell/µl [95% CI −793.20; −425.18], −518.68 cell/µl [95% CI −820.24; −217.12] and −603.60 cell/µl [95% CI −838.69; −368.51], respectively). Significant heterogeneity was seen in the analysis of studies examining benralizumab and mepolizumab. Baseline eosinophilia explained the heterogeneity for mepolizumab (*p* < .001) and benralizumab (*p* < .001) (Tables 1, 2, 3; Table S9).

### 3.9 Change in FeNO

FeNO was assessed in patients treated with mepolizumab and benralizumab. Seven studies examined the effect of mepolizumab on FeNO. Moderate‐quality evidence for a reduction in FeNO after treatment with mepolizumab was found.^27^, ^28^, ^32^, ^33^, ^34^, ^35^, ^38^ FeNO was found to be reduced by −14.23 ppb [95% CI −19.71; −8.75]. Three trials examined the effect of benralizumab on FeNO.^22^, ^23^, ^24^ Conversely, low‐quality evidence indicating no significant change in FeNO after treatment with benralizumab was obtained. FeNO was reduced by −14.18 ppb [95% CI −36.54; 8.17]. Significant heterogeneity was seen in the effect size of individuals given benralizumab. However, this was found by regression to be unrelated to baseline blood eosinophils (*p* = .242) (Tables 1, 2, 3; Table S9).

### 3.10 Comparison to RCT data

**FIGURE 2:**
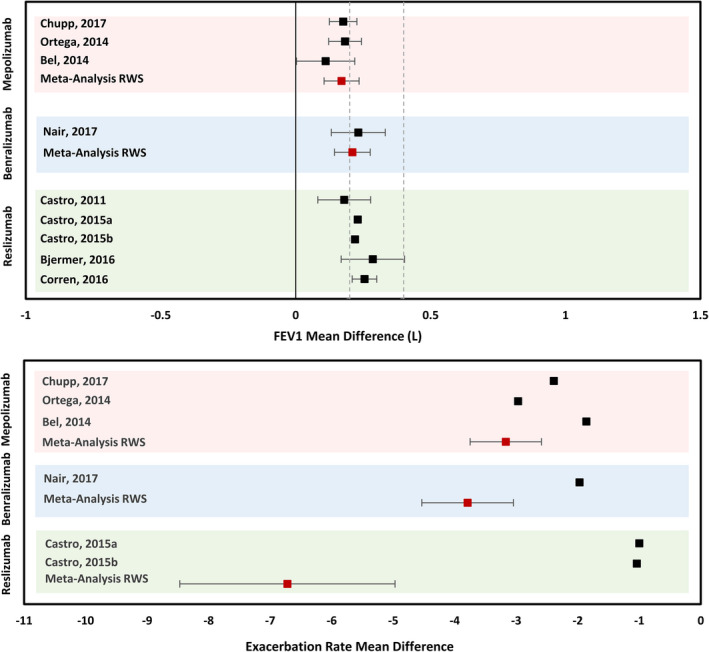
Comparison between meta‐analysis of real‐world studies and active group randomized controlled trial (RCT) data for changes with biological therapy. Active group only data extracted from biological RCTs in EACCI systematic reviews (black squares) is compared against the meta‐analysis of biological RWS from this review (red squares). Markers placed at 1× and 2× the minimally clinically important difference (MCID). Studies clustered by therapeutic agent. FEV_1_ (forced expiratory volume in one second), RWS (Real‐World Studies). No comparable measures assessing control were identified

In this study, precise effect estimates were derived from real‐world studies. These estimates were comparable to figures derived from the active treatment groups of RCT studies. The increase in FEV_1_ observed in real‐world studies examining mepolizumab of 170 ml was comparable to the range of effects observed in RCTs (range 111–183 ml).^43^, ^44^, ^45^ In real‐world studies, a 210 ml increase was seen in FEV_1_ with benralizumab, and this was similar to the RCT results (range 239–330 ml)^46^, ^47^ (Figure 2). Comparisons were also made with annualized exacerbation rate. The decrease in annualized rate of exacerbation with mepolizumab (3.17) and benralizumab (3.79) was consistent with RCT data (range 1.86–2.97 and 2.57)^43^, ^44^, ^45^, ^46^ but less consistent with reslizumab (6.72 in real‐world studies and 1.06 in RCT data)^48^ (Figure 2). It was not possible to make a direct comparison for asthma control between real‐world studies and RCT data owing to differences in measurement methodology (Tables 1, 2, 3; Table S10).

### 3.11 Effect of dupilumab

One study examined the effect of dupilumab on key outcomes.^40^ Owing to the limited number of studies, no meta‐analysis was possible, and results were tabulated (Table 4).

**TABLE 4:** Summary of results for dupilumab

Outcome	No. of participants evaluated for an outcome	Certainty of the evidence (GRADE)	Median [IQR]
ΔAnnualized rate of asthma exacerbation	41 Participants	N/A	−3 [−5 to −2]
ΔACT score	32 Participants	N/A	9 [5 – 12]
ΔOral steroid dose (mg)	37 Participants	N/A	−13 [−20 to −5]
ΔFEV_1_ (L)	39 Participants	N/A	0.2 [−0.3 to 0.62]

Note

Results reflect median estimate provided by the single eligible study examining treatment with dupilumab.^40^

Abbreviations: ACT, asthma control test (higher scores indicating better control); CI, confidence interval; FEV_1_, forced expiratory volume in one second; FeNO, fractional exhaled nitric oxide; IQR, interquartile range.

## 4 DISCUSSION

### 4.1 Summary of main findings

This systematic review provides evidence about the real‐world effects of anti‐IL5 biologicals in asthma. There was moderate certainty of evidence that three biologicals, mepolizumab, benralizumab and reslizumab, decrease the annualized rate of exacerbation and moderate evidence for a decrease in FeNO with mepolizumab. There was low certainty of evidence that the use of mepolizumab and benralizumab in a real‐world environment was associated with an improvement in asthma control as measured by ACT. There was low certainty of evidence for improvement in FEV_1_ with mepolizumab and benralizumab. However, the data suggest that benralizumab has no effect on FeNO. In this study, evidence of a decrease in blood eosinophilia for mepolizumab, benralizumab and reslizumab was observed but with very low certainty.

In this study, benralizumab and mepolizumab were demonstrated to have differing effects on clinical parameters including FENO, FEV_1_ and exacerbations. These should be considered alongside the data from previous studies.^20^, ^49^ As in previous RCT studies, the data provided in this meta‐analysis suggest that mepolizumab, benralizumab and reslizumab have the capacity to reduce the number of exacerbations.^43^, ^44^, ^46^, ^50^, ^51^, ^52^, ^53^, ^54^ Likewise, these have shown that FEV_1_ is improved by treatment with biologicals.^43^, ^44^, ^46^, ^50^, ^51^, ^52^, ^54^ However, unlike previous work, real‐world trials appear to demonstrate that benralizumab has an effect on FEV_1_ which is above the MCID whilst mepolizumab causes a statistically significant change which is below the MCID.^20^ Interestingly, despite the effect on FEV_1_, in this study, benralizumab exerts no clear effect on FeNO. This effect has not been replicated in the context of systematic reviews of RCTs.^20^ In patients with severe eosinophilic asthma with a FeNO‐high phenotype, the use of benralizumab has been associated with a fall in FeNO.^55^ However, this does not occur in individuals with a FeNO‐low phenotype.^56^ Further studies are required to assess the different phenotypes in context of treatment with these monoclonals. This may reflect subtle differences in the mechanism of action between mepolizumab and benralizumab or perhaps infer population effects not seen in RCTs. These differences maybe important to healthcare providers when determining which biological therapy to select for patients with severe asthma. However, these conclusions are based on data from a limited number of participants, for example in the case of the effect of benralizumab on FeNO, a sample of 179 participants over three studies with even less for reslizumab.

As expected, real‐world studies utilized slightly different methodologies for selecting participants which are typically derived from internationally accepted systems, introducing some inherent heterogeneity. However, by synthesizing data, it is possible to get an estimate of the effect across a broad spectrum of clinical practice. Study methodologies also varied, most though were retrospective and a sensitivity analysis restricted to those retrospective studies provided a similar result (Table S17). The methodological variation may have introduced a small amount of heterogeneity. These differences should be taken into consideration when forming conclusions. However, the data suggest that real‐world studies can help to provide a more comprehensive understanding of biologics in real clinical practice.

### 4.2 Areas for further development

In this review, one real‐world study on dupilumab was identified precluding further efforts to perform a meta‐analysis. Further assessment of this agent in real‐world studies is suggested to provide precise effect estimates.

### 4.3 Strengths and limitations

This systematic review and meta‐analysis of data regarding recently licenced biologicals in a real‐world environment provides some of the first estimates of the effect of newer biologicals in real clinic patients. A key strength of the data presented in this study is that there is consistency in effect across primary studies, and that many of the effects observed were significantly above the no‐effect line. Importantly, these outcomes were some of the most widely used in clinical practice. FEV_1_, for example, has been linked to respiratory hospitalization, healthcare costs and mortality amongst asthmatics.^57^, ^58^, ^59^

Nevertheless, there are some limitations to this systematic review which should be considered when interpreting the results. One limitation was the heterogeneity found in the analysis of some outcomes. Although many studies had narrow confidence intervals, heterogeneity was identified between studies. However, there was a consistent direction of effect found amongst studies, and in most, the effects were far above the no‐effect line. Some of the heterogeneity surrounding the effect size between studies was explained by the baseline level of blood eosinophilia. Differences in study populations and endotypes of asthma may have some effect, but there were insufficient analyses to explore this, which prevented us from presenting effect sizes for different endotypes. Methodological variation may have also contributed to the heterogeneity seen. One critical feature is that real‐world studies reflect real clinical environments, and these environments may be different to one another. As such, heterogeneity is to be expected. Conversely, the limited data available for agents like reslizumab artificially reduced some of the observed heterogeneity. However, through synthesis, broad consensus can be obtained.

Many of the included studies are observational and as expected they are at higher risk of bias. This is reflected in the assessment of risk of bias for the included studies. Some of this risk of bias is derived from the methodology used. In this meta‐analysis, most of the studies were retrospective. These studies are an efficient way of exploring the effects of biologicals on severe asthma. However, the presence of these studies has the potential to introduce bias into the analysis particularly via recall bias. This needs to be considered when drawing conclusions from this meta‐analysis. However, the results are in line with RCTs which are not subject to such bias, suggesting that the bias present is insufficient to significantly impair the assessment of outcomes.

This meta‐analysis focused on real‐world studies, in which it can be difficult to account for the placebo effect and for regression to the mean. These issues are inherent when reviewing, assessing, and synthesizing data from real‐world trials. However, we have overcome this issue in this systematic review by comparing results from the active arm of equivalent RCTs, thereby providing external validation of our results. These comparisons demonstrate significant concordance between real‐world studies and RCTs when examining objective measures which has implications for researchers examining these variables and implies that data derived from RCTs are applicable to the broad spectrum of patients seen in clinical practice.

### 4.4 Implications for practice and research

We demonstrate that effect estimates from real‐world studies of benralizumab, mepolizumab and reslizumab are similar to those derived from RCTs confirming that these therapeutics are also effective in typical clinical patients with severe asthma. Effect estimates generated in this study can be used to optimize patient services. However, there are areas where further targeted work could be impactful. For instance, given that reducing OCS use is a major factor in the use of biologicals in clinical practice, gaining further insight into this parameter represents a priority for future studies.

To allow future meta‐analysis to create more precise estimates, we suggest that studies report the following: (1) how patients were determined to have severe asthma, (2) how they were determined to have eosinophilia, (3) details of exacerbation rate, asthma control, quality of life and changes in FEV_1_ (in litres) in terms of mean difference alongside the standard error of the difference and (4) the numbers of patients who no longer require oral steroids after treatment.

The biologicals reviewed in this study showed a significant improvement in several key clinical parameters in a real‐life environment. We argue that to produce a comprehensive profile for biologicals, it is essential for data from real‐world studies, RCTs and registries to be combined and analyzed. Additionally, we need more studies examining the impact of biologicals in different asthma endotypes. Furthermore, in both real‐world studies and RCTs, there are very limited data for the use of these newer biologicals in the paediatric populations, and this remains as a key area for future research.^20^

## 5 CONCLUSION

This meta‐analysis shows that in real‐world studies mepolizumab, benralizumab and reslizumab are effective treatments for asthma when looking at key clinical parameters. The effects observed in real‐world trials are similar to those seen in the active group of equivalent RCTs. Further research is required to provide precise effect estimates for dupilumab in a real‐world setting.

## CONFLICT OF INTEREST

The authors know of no conflict of interest.

## Supporting information

Fig S1

Fig S2

Fig S3

Fig S4

Fig S5

Fig S6

Fig S7

Fig S8

Fig S9

Fig S10

Fig S11

Fig S12

Fig S13

Fig S14

Fig S15

Fig S16

Fig S17

Fig S18

Fig S19

Fig S20

Fig S21

Fig S22

Table S1

Table S2

Table S3

Table S4

Table S5

Table S6

Table S7

Table S8

Table S9

Table S10

Table S11

Table S12

Table S13

Table S14

Table S15

Table S16

Table S17

Supplementary Material

## Data Availability

The data that support the findings of this study are available from the corresponding author upon reasonable request.
