# Supplementary material for: Real‐world efficacy of treatment with benralizumab, dupilumab, mepolizumab and reslizumab for severe asthma: A systematic review and meta‐analysis
Source: Clin Exp Allergy. 2022 Mar 9;52(5):616–27. doi: 10.1111/cea.14112 (PMC9311192; doi:10.1111/cea.14112)
Supplement: Supplementary file 23 — Table S1 [file CEA-52-616-s002.docx]

**Supplementary Table 2: Studies Characteristics of Studies used in Analysis of Mepolizumab**

| Author, Year | N | Age Range | Population | Intervention | Time | Key Outcomes Assessed | Key Biomarkers Assessed | Exacerbation Definition | Adverse Events | Risk of Bias |
| --- | --- | --- | --- | --- | --- | --- | --- | --- | --- | --- |
| Bagnasco, 2019 (25) | 138 | 58  (SD +/- 10) | Severe Eosinophilic Asthma   - ERS/ATS Definition - > 300 cells/μL during the last 12 months year or more than 150 cells/μL before the 1^st^ injection | Mepolizumab | 52 weeks | - Asthma Control: ACT - Exacerbation - Steroid Dosage - Hospitalisation | - FeNO - Blood Eosinophils - FEV1 | - Based on international Definition: ATS/ERS | - Adverse Events Reported in 10% of patients | Moderate |
| Cameli, 2020 (26) | 26 | 56.4  (SD +/- 11.7) | Severe Eosinophilic Asthma   - ERS/ATS Definition - ≥ 300 cells/μL | Mepolizumab | 26 weeks | - Asthma Control: ACT - Exacerbation - Steroid Dosage | - FeNO - Blood Eosinophils - FEV1 | - Based on international Definition: ATS/ERS | - Adverse Events Reported in 50% of patients | Moderate |
| Caminati, 2019 (27) | 69 | 55.4  (SD +/- 11.9) | Severe Eosinophilic Asthma   - ERS/ATS Definition - ≥ 300 cell/μL within the last 12 months and >150 cells/μL when recruited | Mepolizumab | 26 weeks | - Asthma Control: ACT - Steroid Dosage | - FeNO - Blood Eosinophils - FEV1 | - N/A | - No Adverse Events Reported | Moderate |
| Farah, 2019 (28)* | 28 | 60  (SD +/- 16) | Severe Eosinophilic Asthma   - Physician Defined - >300 cell/μL or 150 cells/μL if on maintenance oral steroids | Mepolizumab | 26 weeks | - Asthma Control: ACQ-5 | - FeNO - FEV1 | - N/A | - Not Reported | Moderate |
| Kallieri, 2020 (29)* | 140 | 56  (SD +/- 13) | Severe Eosinophilic Asthma   - ERS/ATS Definition - >300 cell/μL or 150 cells/μL as per official indications | Mepolizumab | 78 weeks | - Asthma Control: ACT - Exacerbation - Steroid Dosage | - Blood Eosinophils - FEV1 | - Based on need for systemic CS or Hospital intervention | - Adverse Events Reported in 27% of patients | Moderate |
| Kavanagh, 2020 (30) | 106 | 53.5  (SD +/- 13.2) | Severe Eosinophilic Asthma   - ERS/ATS Definition - Eosinophils > 300 cells/ μL in the past 12 months | Mepolizumab | 52 weeks | - Asthma Control: ACQ-6 - Exacerbation - Quality of Life: AQLQ | - FeNO - Blood Eosinophils - FEV1 | - Based on need for systemic CS or Hospital intervention | - Not Reported | Low |
| Kotisalmi, 2020 (23) | 64 | 56  (SD +/- 9.75) | Severe Eosinophilic Asthma   - Physician Defined | Benralizumab  (n = 5)  Mepolizumab  (n = 24)  Reslizumab  (n = 13)  Omalizumab | 52 weeks | - Asthma Control: ACT - Exacerbation - Steroid Dosage | - Blood Eosinophils - FEV1 | - Not Stated | - Not Reported | Moderate |
| Numata, 2020 (32) | 24 | 56.5  (SD +/- 12.7) | Severe Eosinophilic Asthma   - Physician Defined - Eosinophil count > 150 or previously eosinophil count >300 cells/μL | Mepolizumab | 52 weeks | - Asthma Control: ACT - Steroid Dosage - Exacerbation | - FeNO - Blood Eosinophils - FEV1 | - Based on need for systemic CS or Hospital intervention | - Not Reported | Moderate |
| Numata, 2019 (31) | 28 | 56.3  (SD +/- 11.8) | Severe Eosinophilic Asthma   - Global Initiative of Asthma (GINA) guideline - Eosinophil count of > 150 cells/μL | Mepolizumab | 48 weeks | - Asthma Control: ACT - Exacerbation - Steroid Dosage | - FeNO - Blood Eosinophils - FEV1 | - Based on need for systemic CS or Hospital intervention | - Not Reported | Moderate |
| Pelaia, 2020 (33) | 88 | 54.51  (SD +/-10.80) | Severe Eosinophilic Asthma   - ERS/ATS Definition - > 300 cells/μl | Mepolizumab | 52 weeks | - Asthma Control: ACT - Exacerbation - Steroid Dosage | - FeNO - Blood Eosinophils - FEV1 | - Not Stated | - No Adverse Events Reported | Moderate |
| Schleich, 2020 (35)* | 116 | 54  (SD +/- 14) | Severe Eosinophilic Asthma   - ERS/ATS Definition | Mepolizumab | 130 weeks | - Asthma Control: ACT + ACQ - Exacerbation - Steroid Dosage - Quality of Life: AQLQ | - FeNO - FEV1 | - Based on need for systemic CS or Hospital intervention | - Not Reported | High |
| Sposato, 2020 (34) | 134 | 58.3  (SD +/- 11) | Severe Eosinophilic Asthma   - ERS/ATS Definition - > 300 cells/μL | Mepolizumab | 48 weeks | - Asthma Control: ACT - Exacerbation | - FeNO - Blood Eosinophils - FEV1 | - Based on need for systemic CS or Hospital intervention | - Not Reported | Low |
| Strauss, 2018 (36) | 36 | 57.88  (SD +/- 11.23) | Severe Eosinophilic Asthma   - Physician Definition - > 150 cells/μL | Mepolizumab | Up to 60 weeks | - Asthma Control: ACQ - Exacerbation | - Blood Eosinophils | - Based on need for systemic CS or Hospital intervention | - Adverse Events Reported in 5.6% patients | Moderate |
| Van Toor, 2020 (37) | 78 | 54  (SD +/- 15) | Severe Eosinophilic Asthma   - Physician Diagnosed - >0.30 cells/μL when on high dose inhaled glucocorticoids or >0.15 cells/μL when on mOCS | Mepolizumab | 52 weeks | - Asthma Control: ACQ - Exacerbation - Hospitalization | - FeNO - FEV1 | - Based on need for systemic CS or Hospital intervention | - Adverse Events Reported in 42% of patients | Low |

Risk of bias for each study assessed using the CASP tool. Grade analysis automatically assumes outcome from observational trial are of low certainty. Data derived from published data and personal communication with authors. FEV1 (forced expiratory volume in one Second), FeNO (fractional exhaled nitric oxide), ACT (Asthma Control Test), ACQ (Asthma Control Questionnaire), AQLQ (Asthma Quality of Life Questionnaire), ERS (European Respiratory Society), ATS (American Thoracic Society), SD (standard deviation), mOCS (maintenance oral corticosteroids steroids). CS (Corticosteroids). All studies were retrospective except for the few prospective ones marked with an *.
