# Supplementary material for: Real‐world efficacy of treatment with benralizumab, dupilumab, mepolizumab and reslizumab for severe asthma: A systematic review and meta‐analysis
Source: Clin Exp Allergy. 2022 Mar 9;52(5):616–27. doi: 10.1111/cea.14112 (PMC9311192; doi:10.1111/cea.14112)
Supplement: Supplementary file 24 — Table S2 [file CEA-52-616-s011.docx]

**Supplementary Table 3: Study Characteristics of Studies used in Analysis of Reslizumab**

| Author, Year | N | Age Range | Population | Intervention | Time | Key Outcomes Assessed | Key Biomarkers Assessed | Exacerbation Definition | Adverse Events | Risk of Bias |
| --- | --- | --- | --- | --- | --- | --- | --- | --- | --- | --- |
| Ibrahim, 2019 (38) | 26 | 52  (SD +/- 13.5) | Severe Eosinophilic Asthma   - Physician Defined - 400 cell/μL | Reslizumab | 104 weeks | - Asthma Control: ACQ-6 - Exacerbation - Steroid Dosage | - Blood Eosinophils - FEV1 | - Based on international Definition: ATS/ERS | - Adverse Events Reported in 4% of patients | Moderate |
| Kotisalmi, 2020 (23) | 64 | 56  (SD +/- 9.75) | Severe Eosinophilic Asthma   - Physician Defined | Benralizumab  (n = 5)  Mepolizumab  (n = 24)  Reslizumab  (n = 13)  Omalizumab | 52 weeks | - Asthma Control: ACT - Exacerbation - Steroid Dosage | - Blood Eosinophils - FEV1 | - Not Stated | - Not Reported | Moderate |

Risk of bias for each study assessed using the CASP tool. Grade analysis automatically assumes outcome from observational trial are of low certainty. Data derived from published data and personal communication with authors. FEV1 (forced expiratory volume in one Second), FeNO (fractional exhaled nitric oxide), ACT (Asthma Control Test), ACQ (Asthma Control Questionnaire), SD (standard deviation).
