# Supplementary material for: Real‐world efficacy of treatment with benralizumab, dupilumab, mepolizumab and reslizumab for severe asthma: A systematic review and meta‐analysis
Source: Clin Exp Allergy. 2022 Mar 9;52(5):616–27. doi: 10.1111/cea.14112 (PMC9311192; doi:10.1111/cea.14112)
Supplement: Supplementary file 25 — Table S3 [file CEA-52-616-s021.docx]

**Supplementary Table 4: Study Characteristics of Studies used in Analysis of Dupilumab**

| Author, Year | N | Age Range | Population | Intervention | Time | Key Outcomes Assessed | Key Biomarkers Assessed | Exacerbation Definition | Adverse Events | Risk of Bias |
| --- | --- | --- | --- | --- | --- | --- | --- | --- | --- | --- |
| Dupin, 2020 (39) | 62 | Median 51 (IQR 44-61) | - Severe Asthma Severe Asthma (non-eosinophilic, non-atopic) | Dupilumab | 52 weeks | - Asthma Control: ACT - Exacerbation - Steroid Dosage | - FEV1 | - Not Stated | - Adverse Events Reported in 28% of patients | Moderate |

Risk of bias for each study assessed using the CASP tool. Grade analysis automatically assumes outcome from observational trial are of low certainty. Data derived from published data and personal communication with authors. FEV1 (forced expiratory volume in one Second), FeNO (fractional exhaled nitric oxide), ACT (Asthma Control Test), ACQ (Asthma Control Questionnaire), SD (standard deviation).
