# Supplementary material for: Real‐world efficacy of treatment with benralizumab, dupilumab, mepolizumab and reslizumab for severe asthma: A systematic review and meta‐analysis
Source: Clin Exp Allergy. 2022 Mar 9;52(5):616–27. doi: 10.1111/cea.14112 (PMC9311192; doi:10.1111/cea.14112)
Supplement: Supplementary file 26 — Table S4 [file CEA-52-616-s006.docx]

**Supplementary Table 5: Grade Assessment for Benralizumab Outcomes**

| **Outcome** | **ROB** | **Imprecision** | **Inconsistency** | **Indirectness** | **Publication Bias** | **Size of Effect** | **Dose Response** | **Confounding change effect** | **Overall** |
| --- | --- | --- | --- | --- | --- | --- | --- | --- | --- |
| **Δ Annualised Rate of Asthma Exacerbation** | Not Serious (0)  3 Studies   - 2 Moderate - 1 Low | Not Serious (0)   - Small CI - Good magnitude | Not Serious (0)   - Point Estimate Similar - Overlapping CI - Consistent Direction of Effect - I^2^ = Minimal | Not Serious (0)   - Applicable Population - Applicable Intervention - Not surrogate outcome - Sufficient Timeframe | Serious (-1)   - Funnel Plot Asymmetrical - Systematic Search performed - Grey Literature Search | Very Large Effect (+2) | N/A | None | Moderate |
| **Δ ACT Score** | Not Serious (0)  4 Studies   - 4 Moderate | Not Serious (0)   - Wide Size CI - Good magnitude | Not Serious (0)   - Point Estimate Similar - Overlapping CI - Consistent Direction of Effect - I^2^ = Substantial | Not Serious (0)   - Applicable Population - Applicable Intervention - Not surrogate outcome - Sufficient Timeframe | Serious (-1)   - Funnel Plot Asymmetrical - Systematic Search performed - Grey Literature Search | Effect Not Large (0) | N/A | None | Very Low |
| **Δ ACQ-6 Score** | Not Serious (0)  2 Studies  - 2 Low | Not Serious (0)   - Wide Size CI - Good magnitude | Not Serious (0)   - Point Estimate Similar - Overlapping CI - Consistent Direction of Effect   I^2^ = Substantial | Not Serious (0)   - Applicable Population - Applicable Intervention - Not surrogate outcome   Sufficient Timeframe | Serious (-1)   - Funnel Plot Asymmetrical - Systematic Search performed   Grey Literature Search | Effect Not Large (0) | N/A | None | Very Low |
| **Δ Oral Steroid Dose** | Not Serious (0)  4 Studies   - 3 Moderate - 1 Low | Not Serious (0)   - Wide CI - Good magnitude | Serious (-1)   - Point Estimate Similar - Overlapping CI - Consistent Direction of Effect - I^2^ = Considerable | Not Serious (0)   - Applicable Population - Applicable Intervention - Not surrogate outcome - Sufficient Timeframe | Serious (-1)   - Funnel Plot Asymmetrical - Systematic Search performed - Grey Literature Search | Effect Not Large (0) | N/A | None | Very Low |
| **Δ FEV1** | Not Serious (0)  5 Studies   - 4 Moderate - 1 Low | Not Serious (0)   - Moderate Size CI - Good magnitude | Not Serious (0)   - Point Estimate Similar - Overlapping CI - Consistent Direction of Effect - I^2^ = Substantial | Not Serious (0)   - Applicable Population - Applicable Intervention - Not surrogate outcome - Sufficient Timeframe | Not Serious (0)   - Funnel Plot Asymmetrical - Systematic Search performed - Grey Literature Search | Effect Not Large (0) | N/A | None | Low |
| **Δ FeNO** | Not Serious (0)  3 Studies   - 2 Moderate - 1 Low | Not Serious (0)   - Wide Size CI - Good magnitude | Serious (-1)   - Point Estimate Similar - Overlapping CI - Consistent Direction of Effect   I^2^ = Considerable | Not Serious (0)   - Applicable Population - Applicable Intervention - Not surrogate outcome - Sufficient Timeframe | Not Serious (0)   - Funnel Plot Reasonable Symmetrical - Systematic Search performed - Grey Literature Search | Large Effect (+1) | N/A | None | Low |
| **Δ Blood Eosinophils** | Not Serious (0)  5 Studies   - 4 Moderate - 1 Low | Not Serious (0)   - Wide CI - Good magnitude | Serious (-1)   - Point Estimate Not Similar - Overlapping CI - Consistent Direction of Effect   I^2^ = Considerable | Not Serious (0)   - Applicable Population - Applicable Intervention - Not surrogate outcome - Sufficient Timeframe | Serious (-1)   - Funnel Plot Asymmetrical - Systematic Search performed - Grey Literature Search | Effect Not Large (0) | N/A | None | Very Low |

FEV1 (forced expiratory volume in one Second), FeNO (fractional exhaled nitric oxide), ACT (Asthma Control Test), ACQ (Asthma Control Questionnaire), CI (Confidence Interval).
