# Supplementary material for: Real‐world efficacy of treatment with benralizumab, dupilumab, mepolizumab and reslizumab for severe asthma: A systematic review and meta‐analysis
Source: Clin Exp Allergy. 2022 Mar 9;52(5):616–27. doi: 10.1111/cea.14112 (PMC9311192; doi:10.1111/cea.14112)
Supplement: Supplementary file 29 — Table S7 [file CEA-52-616-s037.docx]

**Supplementary Table 8: Risk of Bias Assessment for Identified Studies**

| **Name of author** | **Was the cohort recruited in an acceptable way?** | **Was the exposure accurately measured to minimise bias?** | **Was the outcome accurately measured to minimise bias?** | **Have the authors identified all important confounding factors?** | **Have they taken account of the confounding factors in the design and/or analysis?** | **Was the follow up of subjects complete enough?** | **Was the follow up of subjects long enough?** | **Precision** | **Overall ROB** |
| --- | --- | --- | --- | --- | --- | --- | --- | --- | --- |
| **Bagnasco** | ✓ | ✓ | ✓ | ? | ? | ✓ | ? | ✓ | Moderate |
| **Bagnasco** | ✓ | ✓ | ? | ✓ | ✓ | ✓ | ✓ | ✓ | Moderate |
| **Cameli** | ✓ | ✓ | ✓ | ? | ? | ✓ | ? | ✓ | Moderate |
| **Caminati** | ✓ | ✓ | ✓ | ? | ? | ✓ | ? | ✓ | Moderate |
| **Farah** | ✓ | ✓ | ✓ | ? | ? | ✓ | ? | ✓ | Moderate |
| **Ibrahim** | ✓ | ✓ | ✓ | X | ? | ✓ | ✓ | ✓ | Moderate |
| **Kallieri** | ✓ | ✓ | ? | ? | X | ✓ | ✓ | ✓ | Moderate |
| **Kavanagh** | ✓ | ✓ | ✓ | ✓ | ✓ | ✓ | ✓ | ✓ | Low |
| **Kotisalmi** | ✓ | ✓ | ✓ | ✓ | ? | ✓ | ✓ | X | Moderate |
| **Numata** | ✓ | ✓ | ? | ✓ | ✓ | ✓ | ✓ | X | Moderate |
| **Numata** | ✓ | ✓ | ✓ | ✓ | ✓ | ✓ | ? | X | Moderate |
| **Numata** | ✓ | ✓ | ✓ | ✓ | ? | ✓ | ? | X | Moderate |
| **Padilla-Galo** | ✓ | ✓ | ✓ | ✓ | ? | ✓ | ? | ✓ | Moderate |
| **Pelaia** | ✓ | ✓ | ✓ | ✓ | ✓ | ✓ | ? | X | Moderate |
| **Pelaia** | ✓ | ✓ | ✓ | ✓ | ? | ✓ | ✓ | ✓ | Moderate |
| **Schleich** | ? | ? | ✓ | ? | ? | ✓ | ✓ | ✓ | High |
| **Sposatoa** | ✓ | ✓ | ✓ | ✓ | ✓ | ✓ | ✓ | ✓ | Low |
| **Strauss** | ✓ | ✓ | ✓ | ✓ | ? | ✓ | ✓ | ✓ | Moderate |
| **Van Toor** | ✓ | ✓ | ✓ | ✓ | ✓ | ✓ | ✓ | ✓ | Low |
| **Kavanagh** | ✓ | ✓ | ✓ | ✓ | ✓ | ✓ | ✓ | ✓ | Low |

✓ - Yes, X - No, ? – Unclear.
