# Supplementary material for: Real‐world efficacy of treatment with benralizumab, dupilumab, mepolizumab and reslizumab for severe asthma: A systematic review and meta‐analysis
Source: Clin Exp Allergy. 2022 Mar 9;52(5):616–27. doi: 10.1111/cea.14112 (PMC9311192; doi:10.1111/cea.14112)
Supplement: Supplementary file 30 — Table S8 [file CEA-52-616-s010.docx]

**Supplementary Table 9: Exploration of Heterogeneity by Meta regression**

| **Variable** | **Drug** | **Heterogeneity** | **Co-efficient (SE)** | **P-Value** | **95% Confidence Interval** |
| --- | --- | --- | --- | --- | --- |
| Exacerbation Rate | Mepolizumab | 83.62% | -0.0017423 (+/- 0.0009) | 0.063 | -0.004 to 0.0000* |
|  | Benralizumab | 27.97% | -0.00133 (+/- 0.0009) | 0.155 | -0.003 to -0.0005* |
|  | Reslizumab | 0.00% | -0.0013333 (+/- 0.0152) | 0.930 | -0.0284 to -0.0310* |
| FEV1 | Mepolizumab | 12.20% | -0.00003 (+/-0.0001) | 0.828 | -0.0003 to 0.0003* |
|  | Benralizumab | 84.76% | 0.0006703 (+/- 0.0002) | **0.003** | 0.0002 to 0.00111* |
| FENO | Mepolizumab | 16.41% | -0.0234 (+/- 0.012) | **0.046** | -0.046 to -0.0039 |
|  | Benralizumab | 95.23% | -0.0610104 (+/- 0.5212) | 0.242 | -0.1631 to 0.04114* |
| Control (ACT) | Mepolizumab | 79.50% | 0.0016 (+/- 0.003) | 0.544 | -0.0035 to 0.0067* |
|  | Benralizumab | 88.39% | 0.0106 (+/- 0.003) | **0.000** | 0.0051 to 0.0159* |
| Control (ACQ-6) | Mepolizumab | 0.00% | -0.0017 (+/- 0.007) | 0.808 | -0.015 to 0.012* |
| Eosinophil Reduction | Mepolizumab | 91.73% | -0.815 (+/- 0.09) | **0.000** | -1.00 to -0.640 |
|  | Benralizumab | 96.12% | -1.136 (+/- 0.113) | **0.000** | -1.356 to -0.916* |
|  | Reslizumab | 44.00% | -0.72727 (+/-1.5790) | 0.645 | -3.4802 to 1.8250 |
| Steroid Dosage | Mepolizumab | 87.30% | -0.00045 (+/- 0.004) | 0.909 | -0.008 to 0.007* |
|  | Benralizumab | 94.84% | -0.0173 (+/- 0.0114) | 0.129 | -0.04 to 0.0050* |
|  | Reslizumab | 0.00% | -.00281 (+/- 0.0049) | 0.567 | -0.0125 to 0.0068* |

A negative co-efficient indicates a decrease in the asthma output variable equivalent to the co-efficient per unit increase in the eosinophil count. SE (Standard Error), FEV1 (forced expiratory volume in one Second), FeNO (fractional exhaled nitric oxide), ACT (Asthma Control Test), ACQ (Asthma Control Questionnaire).
