# Supplementary material for: Real‐world efficacy of treatment with benralizumab, dupilumab, mepolizumab and reslizumab for severe asthma: A systematic review and meta‐analysis
Source: Clin Exp Allergy. 2022 Mar 9;52(5):616–27. doi: 10.1111/cea.14112 (PMC9311192; doi:10.1111/cea.14112)
Supplement: Supplementary file 31 — Table S9 [file CEA-52-616-s019.docx]

**Supplementary Table 10: Change in Asthma outcome parameter with the Active group in Randomised Control Trial Data**

| **Drug** | **Paper** | **Number** | **Details** | **FEV1** | | **Asthma Control** | | | **Exacerbation** | |
| --- | --- | --- | --- | --- | --- | --- | --- | --- | --- | --- |
|  |  |  |  | **Change** | **CI** | **Test** | **Change** | **SE** | **Change** | **SE** |
| Mepolizumab | Chupp, 2017 ^(41)^ | 269 | S/C Mepolizumab every 4 weeks | 0.176 L | 0.051 | - | - | - | 2.39 | - |
|  | Ortega, 2014^(42)^ | 194 | S/C Mepolizumab every 4 weeks | 0.183 L | 0.061 | ACQ-5 | -0.94 | 0.07 | 2.97 | - |
|  | Bel, 2014^(43)^ | 69 | S/C Mepolizumab every 4 weeks | 0.111 L | 0.11 | - | - | - | 1.86** | - |
| Benralizumab | Nair, 2017^(44)^ | 73 | S/C Benralizumab every 4 week for 3 months then every 8 weeks | 0.23 L | 0.10** | - | - | - | 1.97 | - |
|  |  |  |  |  |  |  |  |  |  |  |
| Reslizumab | Castro, 2011^(54)^ | 53 | IV infusion of Reslizumab every 4 weeks | 0.18 L | 0.10 | ACQ | -0.853 | 0.12 | - | - |
|  |  |  |  |  |  | ACQ-5 | -0.787 | - | - | - |
|  |  |  |  |  |  | ACQ-6 | -0.838 | - | - | - |
|  | Bjermer, 2016^(49)^ | 106 | IV infusion of Reslizumab every 4 weeks | 0.286 L | 0.12 | ACQ-7 | -0.838 | 0.05 | - | - |
|  | Corren, 2016^(50)^ | 396 | IV infusion of Reslizumab every 4 weeks | 0.255 L | 0.05 | ACQ-7 | -0.844 | - | - | - |
|  | Castro, 2015*^(46)^ | 245 | Study a: IV infusion of Reslizumab every 4 weeks | 0.24 L | - | ACQ-7 | -0.91 | - | 1 | - |
|  |  | 233 | Study b: IV infusion of Reslizumab every 4 weeks | 0.20 L | - | ACQ-7 | -1.02 | - | 1.04 | - |

Studies identified using a recent systematic review (18). Data from published studies plus additional data from authors. Number represents total number cited in study within active group being examined. * Represents two duplicate multicentre, double-blind, parallel-group placebo controlled trials. FEV1 (forced expiratory volume in one Second), L (litres), ACQ (Asthma Control Questionnaire). ** Values estimated from figures.
