# Supplementary material for: Real‐world efficacy of treatment with benralizumab, dupilumab, mepolizumab and reslizumab for severe asthma: A systematic review and meta‐analysis
Source: Clin Exp Allergy. 2022 Mar 9;52(5):616–27. doi: 10.1111/cea.14112 (PMC9311192; doi:10.1111/cea.14112)
Supplement: Supplementary file 32 — Table S10 [file CEA-52-616-s028.docx]

**Supplementary Table 11: Baseline Eosinophil Level**

| Author, Year | Drug | Pre-Intervention | | | Post-Intervention | |
| --- | --- | --- | --- | --- | --- | --- |
|  |  | Mean | Standard Deviation | | Mean | Standard Deviation |
| Bagnasco, 2020 | Benralizumab | 581 | (SD +/- 556) | 24 | | (SD +/- 48) |
| Numata, 2020 | Benralizumab | 458 | (SD +/- 338) | 0 | | (SD +/- 0) |
| Padillo-Gala, 2020 | Benralizumab | 757.2 | (SD +/- 278) | 15.2 | | (SD +/- 13.6) |
| Kavanagh, 2020 | Benralizumab | 290 | (SD +/- 250) | 70 | | (SD +/- 28) |
| Cameli, 2020 | Mepolizumab | 905.4 | (SD +/- 628.7) | 75 | | (SD +/- 54.8) |
| Caminati, 2019 | Mepolizumab | 983 | (SD +/- 1021.3) | 130.5 | | (SD +/- 192.3) |
| Kallieri, 2020 | Mepolizumab | 703 | (SD +/- 537) | 661 | | (SD +/- 456) |
| Kavanagh, 2020 | Mepolizumab | 290 | (SD +/- 310) | 70 | | (SD +/- 70) |
| Kotisalmi, 2020 | Mepolizumab, Benralizumab, Reslizumab | 450 | (SD +/- 370) | - | | - |
| Numata, 2020 | Mepolizumab | 1228 | (SD +/- 2360) | 99 | | (SD +/- 131) |
| Numata, 2019 | Mepolizumab | 873 | (SD +/- 1851) | 71 | | (SD +/- 64) |
| Pelaia, 2020 | Benralizumab | 947.9 | (SD +/- 602.5) | 0.00 | | (SD +/- 0.00) |
| Pelaia, 2020 | Mepolizumab | 989 | (SD +/- 1082) | 100.1 | | (SD +/- 124.2) |
| Sposato, 2020 | Mepolizumab | 712 | - | - | | - |
| Strauss, 2018 | Mepolizumab | 438 | - | - | | - |
| Van Toor, 2020 | Mepolizumab | 450 | (SD +/- 450) | - | | - |
| Ibrahim, 2019 | Reslizumab | 780 | (SD +/- 510) | 40 | | (SD +/- 30) |

SD (Standard Deviation).
