# Supplementary material for: Real‐world efficacy of treatment with benralizumab, dupilumab, mepolizumab and reslizumab for severe asthma: A systematic review and meta‐analysis
Source: Clin Exp Allergy. 2022 Mar 9;52(5):616–27. doi: 10.1111/cea.14112 (PMC9311192; doi:10.1111/cea.14112)
Supplement: Supplementary file 34 — Table S12 [file CEA-52-616-s036.docx]

**Supplementary Table 13: Funding and Conflicts of Interest**

| Author, Year | Drug | Industry Funding/Declared Conflicts of Interest |
| --- | --- | --- |
| Bagnasco, 2020 (19) | Benralizumab | None Declared |
| Numata, 2020 (21) | Benralizumab | None Declared |
| Padillo-Gala, 2020 (20) | Benralizumab | None Declared |
| Pelaia, 2020 (24) | Benralizumab | None Declared |
| Kavanagh, 2020 (22) | Benralizumab | 3 authors reported advisory board and speaker fees and congress travel support from GSK, Astrazeneca, Chiesi, Napp, and Teva pharmaceuticals. 1 author reported travel support from Teva. 1 author reports speaker fees and congress travel support from AstraZeneca. |
| Kotisalmi, 2020 (23) | Benralizumab  Mepolizumab  Reslizumab | None Declared |
| Bagnasco, 2019 (25) | Mepolizumab | None Declared |
| Cameli, 2020 (26) | Mepolizumab | None Declared |
| Caminati, 2019 (27) | Mepolizumab | None Declared |
| Farah, 2019 (28) | Mepolizumab | None Declared |
| Kallieri, 2020 (29) | Mepolizumab | None Declared |
| Kavanagh, 2020 (30) | Mepolizumab | 1 author reported travel support from Teva. 3 authors reported advisory board and speaker fees and congress travel support from GSK, AstraZeneca, Chiesi, Napp, and Teva pharmaceuticals. 1 author reported speaker fees and congress travel support from Astrazeneca. |
| Kotisalmi, 2020 (23) | Mepolizumab | None Declared |
| Numata, 2020 (32) | Mepolizumab | None Declared |
| Numata, 2019 (31) | Mepolizumab | None Declared |
| Pelaia, 2020 (33) | Mepolizumab | None Declared |
| Schleich, 2020 (35) | Mepolizumab | None Declared |
| Sposato, 2020 (34) | Mepolizumab | None Declared |
| Strauss, 2018 (36) | Mepolizumab | None Declared |
| Van Toor, 2020 (37) | Mepolizumab | Sponsored by a grant from GlaxoSmithKline |
| Ibrahim, 2019 (38) | Reslizumab | The drug used in the study was provided as part of an early access program, but the company had no role in the design, collection, analysis of data or preparation of the manuscript. |
