# Supplementary material for: Real‐world efficacy of treatment with benralizumab, dupilumab, mepolizumab and reslizumab for severe asthma: A systematic review and meta‐analysis
Source: Clin Exp Allergy. 2022 Mar 9;52(5):616–27. doi: 10.1111/cea.14112 (PMC9311192; doi:10.1111/cea.14112)
Supplement: Supplementary file 35 — Table S13 [file CEA-52-616-s031.docx]

**Supplementary Table 14: Funding and Conflicts of Interest: A Sensitivity Analysis**

| Variable | Drug | Sensitivity Analysis Outcome | Overall Outcome |
| --- | --- | --- | --- |
| Δ Exacerbations | Mepolizumab | -3.43 [-3.69, -3.17] | -3.17 [-3.74, -2.59] |
|  | Benralizumab | -3.94 [-5.66, -2.21] | -3.79 [-4.53, -3.04] |
|  | Reslizumab | N/A | -6.72 [-8.47, -4.97] |
| Δ Control | Mepolizumab | +6.15 [5.14, 7.15] | +6.15 [5.14, 7.15] |
|  | Benralizumab | +5.82 [3.39, 8.25] | +5.82 [3.39, 8.25] |
|  | Reslizumab | N/A | N/A |
| Δ FEV1 | Mepolizumab | +0.17 [0.10, 0.25] | +0.17 [0.11, 0.24] |
|  | Benralizumab | +0.24 [0.00, 0.48} | +0.21 [0.08, 0.34] |
|  | Reslizumab | N/A | N/A |
| Δ FeNO | Mepolizumab | -15.43 [-21.27, -9.59] | -14.23 [-19.71, -8.75} |
|  | Benralizumab | -15.28 [-54.90, 24.33] | -14.18 [-36.54, 8.17] |
|  | Reslizumab | N/A | N/A |
| Δ Eosinophils | Mepolizumab | -669.48 [-825.65, -513.50] | -609.19 [-793.20, -425.18] |
|  | Benralizumab | -608.59 [-852.28, -364.89] | -518.68 [-820.24, -217.12] |
|  | Reslizumab | N/A | -603.60 [-838.69, -368.51] |
| Δ Steroids | Mepolizumab | -4.85 [-7.30, -2.39] | -5.30 [-7.50, -3.10] |
|  | Benralizumab | -8.08 [-16.20, 0.05] | -8.35 [-13.83, -2.87] |
|  | Reslizumab | N/A | -3.90 [-5.26, -2.54] |
