# Supplementary material for: Real‐world efficacy of treatment with benralizumab, dupilumab, mepolizumab and reslizumab for severe asthma: A systematic review and meta‐analysis
Source: Clin Exp Allergy. 2022 Mar 9;52(5):616–27. doi: 10.1111/cea.14112 (PMC9311192; doi:10.1111/cea.14112)
Supplement: Supplementary file 36 — Table S14 [file CEA-52-616-s029.docx]

**Supplementary Table 15: Studies at low risk of bias: a sensitivity analysis**

| Variable | Drug | Outcome | Overall Outcome |
| --- | --- | --- | --- |
| Δ Exacerbations | Mepolizumab | -2.80 [-3.78, -1.82] | -3.17 [-3.74, -2.59] |
|  | Benralizumab | N/A | -3.79 [-4.53, -3.04] |
|  | Reslizumab | N/A | -6.72 [-8.47, -4.97] |
| Δ Control | Mepolizumab | N/A | 6.15 [5.14, 7.15] |
|  | Benralizumab | N/A | 5.82 [3.39, 8.25] |
|  | Reslizumab | N/A | N/A |
| Δ FEV1 | Mepolizumab | N/A | +0.17 [0.11, 0.24] |
|  | Benralizumab | N/A | +0.21 [0.08, 0.34] |
|  | Reslizumab | N/A | N/A |
| Δ FeNO | Mepolizumab | -6.59 [-15.12, 2.13] | -14.23 [-19.71, -8.75} |
|  | Benralizumab | N/A | -14.18 [-36.54, 8.17] |
|  | Reslizumab | N/A | N/A |
| Δ Eosinophils | Mepolizumab | -379.37 [-690.51, -68.22] | -609.19 [-793.20, -425.18] |
|  | Benralizumab | N/A | -518.68 [-820.24, -217.12] |
|  | Reslizumab | N/A | -603.60 [-838.69, -368.51] |
| Δ Steroids | Mepolizumab | N/A | -5.30 [-7.50, -3.10] |
|  | Benralizumab | N/A | -8.35 [-13.83, -2.87] |
|  | Reslizumab | N/A | -3.90 [-5.26, -2.54] |
