# Supplementary material for: Real‐world efficacy of treatment with benralizumab, dupilumab, mepolizumab and reslizumab for severe asthma: A systematic review and meta‐analysis
Source: Clin Exp Allergy. 2022 Mar 9;52(5):616–27. doi: 10.1111/cea.14112 (PMC9311192; doi:10.1111/cea.14112)
Supplement: Supplementary file 37 — Table S15 [file CEA-52-616-s033.docx]

**Supplementary Table 16: Retrospective and Prospective Anti-IL5 Biologics Studies**

| **Mepolizumab** | | | **Benralizumab** | | | **Reslizumab** | | |
| --- | --- | --- | --- | --- | --- | --- | --- | --- |
| *Retrospective* | *Prospective* | | *Retrospective* | *Prospective* | | *Retrospective* | *Prospective* | |
| Bagnasco, 2019 (25)  Cameli, 2020 (26)  Caminati, 2019 (27)  Kavanagh, 2020 (30)  Kotisalmi, 2020 (23)  Numata, 2020 (32)  Numata, 2019 (31)  Pelaia, 2020 (33)  Sposato, 2020 (34)  Strauss, 2018 (36)  Van Toor, 2020 (37) | | Farah, 2019 (28)  Kallieri, 2020 (29)  Schleich, 2020 (35) | Bagnasco, 2020 (19)  Numata, 2020 (21)  Pelaia, 2020 (24)  Kavanagh, 2020 (22)  Kotisalmi, 2020 (23)  Padillo-Gala, 2020 (20) | | N/A | Ibrahim, 2019 (38)  Kotisalmi, 2020 (23) | | N/A |
