# Supplementary material for: Real‐world efficacy of treatment with benralizumab, dupilumab, mepolizumab and reslizumab for severe asthma: A systematic review and meta‐analysis
Source: Clin Exp Allergy. 2022 Mar 9;52(5):616–27. doi: 10.1111/cea.14112 (PMC9311192; doi:10.1111/cea.14112)
Supplement: Supplementary file 40 — Supplementary Material [file CEA-52-616-s038.pdf]

Real-World Efficacy of Treatment with Biologicals (Mepolizumab, Benralizumab, Reslizumab and Dupilumab) for Severe Asthma: A Systematic Review and Meta-Analysis

Supplementary Tables, Figures and Methods

Contents:

Supplementary Methods 1: Initial Search strategy for Embase	Page 3
Supplementary Methods 2: Project Overview	Page 5
Supplementary Results 1: Characteristics of excluded papers	Page 5
Supplementary Table 1: Study Characteristics of Studies used in Analysis of Benralizumab	Page 6
Supplementary Table 2: Studies Characteristics of Studies used in Analysis of Mepolizumab	Page 7
Supplementary Table 3: Study Characteristics of Studies used in Analysis of Reslizumab	Page 9
Supplementary Table 4: Study Characteristics of Studies used in Analysis of Dupilumab	Page 10
Supplementary Table 5: Grade Assessment for Benralizumab Outcomes	Page 11
Supplementary Table 6: Grade Assessment for Mepolizumab Outcomes	Page 12
Supplementary Table 7: Grade Assessment for Reslizumab Outcomes	Page 13
Supplementary Table 8: Risk of Bias Assessment for Identified Studies	Page 14
Supplementary Table 9: Exploration of Heterogeneity by Meta regression	Page 15
Supplementary Table 10: Change in Asthma outcome parameter with the Active group in Randomised Control Trial Data	Page 16
Supplementary Table 11: Baseline Eosinophil Level	Page 17
Supplementary Table 12: PRISM Checklist	Page 18
Supplementary Table 13: Funding and Conflicts of Interest	Page 20
Supplementary Table 14: Funding and Conflicts of Interest: A Sensitivity Analysis	Page 21
Supplementary Table 15: Studies using Low Risk of Bias: A Sensitivity Analysis	Page 22
Supplementary Table 16: Retrospective and Prospective Anti-IL5 studies	Page 23
Supplementary Table 17: Retrospective and Prospective Studies: A Sensitivity Analysis	Page 24
Supplementary Figure 1: Mepolizumab Annualised Exacerbation Rate Forest Plot	Page 25
Supplementary Figure 2: Benralizumab Annualised Exacerbation Rate Forest Plot	Page 26
Supplementary Figure 3: Reslizumab Annualised Exacerbation Rate Forest Plot	Page 27
Supplementary Figure 4: Mepolizumab Change in FEV1 Forest Plot	Page 28
Supplementary Figure 5: Benralizumab Change in FEV1 Forest Plot	Page 29
Supplementary Figure 6: Mepolizumab Change in FeNO Forest Plot	Page 30
Supplementary Figure 7: Benralizumab Change in FeNO Forest Plot	Page 31
Supplementary Figure 8: Mepolizumab Change in ACT Forest Plot	Page 32
Supplementary Figure 9: Benralizumab Change in ACT Forest Plot	Page 33
Supplementary Figure 10: Mepolizumab Change in ACQ-6 Forest Plot	Page 34
Supplementary Figure 11: Mepolizumab Change in Eosinophils Forest Plot	Page 35
Supplementary Figure 12: Benralizumab Change in Eosinophils Forest Plot	Page 36
Supplementary Figure 13: Reslizumab Change in Eosinophils Forest Plot	Page 37
Supplementary Figure 14: Mepolizumab Change in Steroid Dosage Forest Plot	Page 38
Supplementary Figure 15: Benralizumab Change in Steroid Dosage Forest Plot	Page 39
Supplementary Figure 16: Reslizumab Change in Steroid Dosage Forest Plot	Page 40
Supplementary Figure 17: Annualised Exacerbation Rate Funnel Plot	Page 41
Supplementary Figure 18: FEV1 Change Funnel Plot	Page 42
Supplementary Figure 19: FeNO Change Funnel Plot	Page 43
Supplementary Figure 20: Change in Control Funnel Plot	Page 44
Supplementary Figure 21: Change in Eosinophils Funnel Plot	Page 45
Supplementary Figure 22: Change in Steroids Funnel Plot	Page 46

Supplementary Methods 1. Initial Search strategy for Embase

Translation for EMBASE

Intervention:

1. mepolizumab.mp. or mepolizumab/

2. nucala.mp.

3. reslizumab.mp. or reslizumab/

4. cinqair.mp.

5. cinqaero.mp.

6. benralizumab.mp. or benralizumab/

7. fasenra.mp.

8. dupilumab.mp. or dupilumab/

9. dupixent.mp.

10. omalizumab.mp. or omalizumab/

11. tralokinumab.mp. or tralokinumab/

12. lebrikizumab.mp. or exp lebrikizumab/

13. TNX-650.mp.

14. tezepelumab.mp. or tezepelumab/

15. brodalumab.mp. or brodalumab/

16. siliq.mp.

17. kyntheum.mp.

18. ligelizumab.mp. or ligelizumab/

19. QGE031.mp.

20. 1 or 2 or 3 or 4 or 5 or 6 or 7 or 8 or 9 or 10 or 11 or 12 or 13 or 14 or 15 or 16 or 17 or 18 or 19

Participants:

21. asthma/ or allergic asthma/ or aspirin exacerbated respiratory disease/ or asthmatic state/ or exercise induced asthma/ or experimental asthma/ or extrinsic asthma/ or intrinsic asthma/ or mild intermittent asthma/ or mild persistent asthma/ or moderate persistent asthma/ or nocturnal asthma/ or occupational asthma/ or severe persistent asthma/

22. asthma*.ti,ab.

23. 21 or 22

Study Design:

24. case control study/

25. longitudinal study/

26. retrospective study/

27. cohort analysis/

28. (Cohort adj (study or studies)).mp.

29. (Case control adj (study or studies)).tw.

30. (follow up adj (study or studies)).tw.

31. (observational adj (study or studies)).tw.

32. (epidemiologic$ adj (study or studies)).tw.

33. Real-World OR Realworld.mp.

34. Real-Life OR reallife.mp.

35. 24 or 25 or 26 or 27 or 28 or 29 or 30 or 31 or 32 or 33 or 34

Study Exclusion Filter

36. editorial/ or review/ or case report/ or case report*.mp.

37. editorial*.mp.

38. conference abstract*.mp.

39. conference paper*.mp. or exp conference paper/ or exp conference abstract/ or exp "conference review"/ or exp symposium/ or exp workshop/

40. ((systematic or narrative) adj2 review*).mp. or "systematic review"/

41. ((("semi-structured" or semistructured or unstructured or informal or "in-depth" or indepth or "face-to-face" or structured or guide) adj3 (interview* or discussion* or questionnaire*)) or ("focus group*" or qualitative or ethnograph* or fieldwork or "field work" or "key informant")).ti,ab.

42. interview/ or information processing/ or verbal communication/ or qualitative research/

43. exp animal model/ or exp biological model/ or exp avian model/ or exp bovine model/ or exp canine model/ or exp caprine model/ or exp equine model/ or exp feline model/ or exp fish model/ or exp frog model/ or exp fruit fly model/ or exp invertebrate model/ or exp nematode model/ or exp ovine model/ or exp porcine model/ or exp primate model/ or exp rabbit model/ or exp rodent model/

44. exp model/ or exp adverse outcome pathway/ or exp anatomic model/ or exp biological model/ or exp disease model/ or exp experimental model/ or exp genetic model/ or exp information model/ or exp lung model/ or exp membrane model/ or exp molecular model/ or exp nonbiological model/ or exp population model/ or exp process model/ or exp simulation/ or exp structural model/ or exp theoretical model/

45. mouse/ or exp murine/ or exp experimental mouse/ or exp mus booduga/ or exp mus musculus/ or exp mus spretus/ or exp mus terricolor/

46. 36 or 37 or 38 or 39 or 40 or 41 or 42 or 43 or 44 or 45

47. 35 not 47

Integration:

48. 20 and 23 and 47

49. limit 48 to English language

Supplementary Methods 2.

Project Overview: This study is one project in a larger body of work with a universal search strategy designed to facilitate comparisons between various individual studies. As per the study protocol, the search strategy encompassed 10 specific biologicals, of which 4 biologicals were assessed in this project. One biological, omalizumab, was excluded from the analysis and remains the subject of future work. The other 5 biologicals had no studies identified. For this analysis, case reports and case series were excluded. Studies were excluded if they had less than 20 participants in total. Post hoc analysis was extended to include data regarding oral corticosteroid dose and FEV1. Adverse events were excluded from this analysis and remains the subject of future work.

Supplementary Results 1:

Characteristics of Excluded Papers: 45 papers were excluded from the analysis after screening (Figure 1). Of these, 8.9% were excluded as a result of including too few participants, 4.4% were excluded for not being written in English, 20% were excluded for assessing the wrong outcome, 31.1% were excluded for assessing the wrong population, 15.6% were excluded as they assessed the wrong question and 20% were excluded for being the wrong publication type. 88.9% of excluded papers (40 studies) examined Anti-IgE therapies, including all papers excluded for not being in English. 11.1% of excluded papers (5 studies) examined Anti-IL5 therapies. Of these, 2 were excluded for being the wrong population, 2 were excluded for examining the wrong outcome and 1 was excluded for examining the wrong question.

Supplementary Results 2:

Sensitivity Analysis: Sensitivity analyses were conducted examining retrospective studies, studies without industry funding and studies with a low risk of bias. All results align with those in the main texted with one exception, no significant difference was observed in FeNO after treatment with mepolizumab when examining low risk of bias studies alone.

Supplementary Table 1: Study Characteristics of Studies used in Analysis of Benralizumab

Author, Year	N	Age Range	Population	Intervention	Time	Key Outcomes Assessed	Key Biomarkers Assessed	Exacerbation Definition	Adverse Events	Risk of Bias
Bagnasco, 2020 (19)	59	57.8 (SD +/- 10.2)	Severe Eosinophilic Asthma<br>• ERS/ATS Definition<br>• 300 cells/µl	Benralizumab	24 weeks	• Asthma Control: ACT<br>• Exacerbation<br>• Steroid Dosage<br>• Hospitalisation	• FeNO<br>• Blood Eosinophils<br>• FEV1	• Based on international Definition: ATS/ERS	• Adverse Events Reported in 15% of patients	Moderate
Numata, 2020 (21)	24	57.5 (SD +/- 13.4)	Severe Eosinophilic Asthma<br>• Physician Defined<br>• 150 cells/µl at baseline or ≥ 300 cells/µl in the previous year	Benralizumab	24 weeks	• Asthma Control: ACT<br>• Steroid Dosage	• FeNO<br>• Blood Eosinophils<br>• FEV1	• Based on need for systemic CS or Hospital intervention	• Adverse Events Reported in 16.7% of patients	Moderate
Padillo-Gala, 2020 (20)	42	53.6 (SD +/- 11.0)	Severe Eosinophilic Asthma<br>• Global Initiative of Asthma (GINA) guidelines<br>• ≥ 300 cells/µL in the previous 12 months or ≥ 150 cells/µL in case of corticosteroid dependence	Benralizumab	26 weeks	• Asthma Control: ACT<br>• Steroid Dosage	• FeNO<br>• Blood Eosinophils<br>• FEV1	• Based on symptoms: Patients trained to identify symptoms and record need for CS	• Adverse Events Reported in 16.7% of patients	Moderate
Pelaia, 2020 (24)	22	58.5 (SD +/- 10.55)	Severe Atopic Eosinophilic Asthma<br>• ERS/ATS Definition<br>• Skin Prick Test Positive<br>• > 300 cells/µl	Benralizumab	24 weeks	• Asthma Control: ACT<br>• Exacerbation<br>• Steroid Dosage	• Blood Eosinophils<br>• FEV1	• Not Stated	• No Adverse Events Reported	Moderate
Kavanagh, 2020 (22)	130	52.8 (SD +/- 14.0)	Severe eosinophilic asthma<br>• ERS/ATS definition<br>• > 300 cells/µl in the preceding 12 months	Benralizumab	48 weeks	• Asthma Control: ACQ-6<br>• Exacerbation<br>• Steroid Dosage<br>• Quality of Life: AQLQ	• FeNO<br>• Blood Eosinophils<br>• FEV1	• Based on need for systemic CS or Hospital intervention	• Adverse Events Reported in 2% of patients	Low
Kotisalmi, 2020 (23)	64	56 (SD +/- 9.75)	Severe Eosinophilic Asthma<br>• Physician Defined	Benralizumab (n = 5)<br>Mepolizumab (n = 24)<br>Reslizumab (n = 13)<br>Omalizumab	52 weeks	• Asthma Control: ACT<br>• Exacerbation<br>• Steroid Dosage	• Blood Eosinophils<br>• FEV1	• Not Stated	• Not Reported	Moderate

Risk of bias for each study assessed using the CASP tool. Grade analysis automatically assumes outcome from observational trial are of low certainty. Data derived from published data and personal communication with authors. FEV1 (forced expiratory volume in one Second), FeNO (fractional exhaled nitric oxide), ACT (Asthma Control Test), ACQ (Asthma Control Questionnaire), AQLQ (Asthma Quality of Life Questionnaire), ERS (European Respiratory Society), ATS (American Thoracic Society), SD (standard deviation). CS (Corticosteroids).

Supplementary Table 2: Studies Characteristics of Studies used in Analysis of Mepolizumab

Author, Year	N	Age Range	Population	Intervention	Time	Key Outcomes Assessed	Key Biomarkers Assessed	Exacerbation Definition	Adverse Events	Risk of Bias
Bagnasco, 2019 (25)	138	58<br>(SD +/- 10)	Severe Eosinophilic Asthma<br>• ERS/ATS Definition<br>• > 300 cells/µL during the last 12 months year or more than 150 cells/µL before the 1st injection	Mepolizumab	52 weeks	• Asthma Control: ACT<br>• Exacerbation<br>• Steroid Dosage<br>• Hospitalisation	• FeNO<br>• Blood Eosinophils<br>• FEV1	• Based on international Definition: ATS/ERS	• Adverse Events Reported in 10% of patients	Moderate
Cameli, 2020 (26)	26	56.4<br>(SD +/- 11.7)	Severe Eosinophilic Asthma<br>• ERS/ATS Definition<br>• ≥ 300 cells/µL	Mepolizumab	26 weeks	• Asthma Control: ACT<br>• Exacerbation<br>• Steroid Dosage	• FeNO<br>• Blood Eosinophils<br>• FEV1	• Based on international Definition: ATS/ERS	• Adverse Events Reported in 50% of patients	Moderate
Caminati, 2019 (27)	69	55.4<br>(SD +/- 11.9)	Severe Eosinophilic Asthma<br>• ERS/ATS Definition<br>• ≥ 300 cell/µL within the last 12 months and >150 cells/µL when recruited	Mepolizumab	26 weeks	• Asthma Control: ACT<br>• Steroid Dosage	• FeNO<br>• Blood Eosinophils<br>• FEV1	• N/A	• No Adverse Events Reported	Moderate
Farah, 2019 (28)*	28	60<br>(SD +/- 16)	Severe Eosinophilic Asthma<br>• Physician Defined<br>• >300 cell/µL or 150 cells/µL if on maintenance oral steroids	Mepolizumab	26 weeks	• Asthma Control: ACQ-5	• FeNO<br>• FEV1	• N/A	• Not Reported	Moderate
Kallieri, 2020 (29)*	140	56<br>(SD +/- 13)	Severe Eosinophilic Asthma<br>• ERS/ATS Definition<br>• >300 cell/µL or 150 cells/µL as per official indications	Mepolizumab	78 weeks	• Asthma Control: ACT<br>• Exacerbation<br>• Steroid Dosage	• Blood Eosinophils<br>• FEV1	• Based on need for systemic CS or Hospital intervention	• Adverse Events Reported in 27% of patients	Moderate
Kavanagh, 2020 (30)	106	53.5<br>(SD +/- 13.2)	Severe Eosinophilic Asthma<br>• ERS/ATS Definition<br>• Eosinophils > 300 cells/ µL in the past 12 months	Mepolizumab	52 weeks	• Asthma Control: ACQ-6<br>• Exacerbation<br>• Quality of Life: AQLQ	• FeNO<br>• Blood Eosinophils<br>• FEV1	• Based on need for systemic CS or Hospital intervention	• Not Reported	Low
Kotisalmi, 2020 (23)	64	56<br>(SD +/- 9.75)	Severe Eosinophilic Asthma<br>• Physician Defined	Benralizumab (n = 5)<br>Mepolizumab (n = 24)<br>Reslizumab (n = 13)	52 weeks	• Asthma Control: ACT<br>• Exacerbation<br>• Steroid Dosage	• Blood Eosinophils<br>• FEV1	• Not Stated	• Not Reported	Moderate

				Omalizumab						
Numata, 2020 (32)	24	56.5<br>(SD +/- 12.7)	Severe Eosinophilic Asthma<br>• Physician Defined<br>• Eosinophil count > 150 or previously eosinophil count >300 cells/µL	Mepolizumab	52 weeks	• Asthma Control: ACT<br>• Steroid Dosage<br>• Exacerbation	• FeNO<br>• Blood Eosinophils<br>• FEV1	• Based on need for systemic CS or Hospital intervention	• Not Reported	Moderate
Numata, 2019 (31)	28	56.3<br>(SD +/- 11.8)	Severe Eosinophilic Asthma<br>• Global Initiative of Asthma (GINA) guideline<br>• Eosinophil count of > 150 cells/µL	Mepolizumab	48 weeks	• Asthma Control: ACT<br>• Exacerbation<br>• Steroid Dosage	• FeNO<br>• Blood Eosinophils<br>• FEV1	• Based on need for systemic CS or Hospital intervention	• Not Reported	Moderate
Pelaia, 2020 (33)	88	54.51<br>(SD +/-10.80)	Severe Eosinophilic Asthma<br>• ERS/ATS Definition<br>• > 300 cells/µl	Mepolizumab	52 weeks	• Asthma Control: ACT<br>• Exacerbation<br>• Steroid Dosage	• FeNO<br>• Blood Eosinophils<br>• FEV1	• Not Stated	• No Adverse Events Reported	Moderate
Schleich, 2020 (35)*	116	54<br>(SD +/- 14)	Severe Eosinophilic Asthma<br>• ERS/ATS Definition	Mepolizumab	130 weeks	• Asthma Control: ACT + ACQ<br>• Exacerbation<br>• Steroid Dosage<br>• Quality of Life: AQLQ	• FeNO<br>• FEV1	• Based on need for systemic CS or Hospital intervention	• Not Reported	High
Sposato, 2020 (34)	134	58.3<br>(SD +/- 11)	Severe Eosinophilic Asthma<br>• ERS/ATS Definition<br>• > 300 cells/µL	Mepolizumab	48 weeks	• Asthma Control: ACT<br>• Exacerbation	• FeNO<br>• Blood Eosinophils<br>• FEV1	• Based on need for systemic CS or Hospital intervention	• Not Reported	Low
Strauss, 2018 (36)	36	57.88<br>(SD +/- 11.23)	Severe Eosinophilic Asthma<br>• Physician Definition<br>• > 150 cells/µL	Mepolizumab	Up to 60 weeks	• Asthma Control: ACQ<br>• Exacerbation	• Blood Eosinophils	• Based on need for systemic CS or Hospital intervention	• Adverse Events Reported in 5.6% patients	Moderate
Van Toor, 2020 (37)	78	54<br>(SD +/- 15)	Severe Eosinophilic Asthma<br>• Physician Diagnosed<br>• >0.30 cells/µL when on high dose inhaled glucocorticoids or >0.15 cells/µL when on mOCS	Mepolizumab	52 weeks	• Asthma Control: ACQ<br>• Exacerbation<br>• Hospitalization	• FeNO<br>• FEV1	• Based on need for systemic CS or Hospital intervention	• Adverse Events Reported in 42% of patients	Low

Risk of bias for each study assessed using the CASP tool. Grade analysis automatically assumes outcome from observational trial are of low certainty. Data derived from published data and personal communication with authors. FEV1 (forced expiratory volume in one Second), FeNO (fractional exhaled nitric oxide), ACT (Asthma Control Test), ACQ (Asthma Control Questionnaire), AQLQ (Asthma Quality of Life Questionnaire), ERS (European Respiratory Society), ATS (American Thoracic Society), SD (standard deviation), mOCS (maintenance oral corticosteroids steroids). CS (Corticosteroids). All studies were retrospective except for the few prospective ones marked with an *.

Supplementary Table 3: Study Characteristics of Studies used in Analysis of Reslizumab

Author, Year	N	Age Range	Population	Intervention	Time	Key Outcomes Assessed	Key Biomarkers Assessed	Exacerbation Definition	Adverse Events	Risk of Bias
Ibrahim, 2019 (38)	26	52 (SD +/- 13.5)	Severe Eosinophilic Asthma<br>• Physician Defined<br>• 400 cell/µL	Reslizumab	104 weeks	• Asthma Control: ACQ-6<br>• Exacerbation<br>• Steroid Dosage	• Blood Eosinophils<br>• FEV1	• Based on international Definition: ATS/ERS	• Adverse Events Reported in 4% of patients	Moderate
Kotisalmi, 2020 (23)	64	56 (SD +/- 9.75)	Severe Eosinophilic Asthma<br>• Physician Defined	Benralizumab (n = 5)<br>Mepolizumab (n = 24)<br>Reslizumab (n = 13)<br>Omalizumab	52 weeks	• Asthma Control: ACT<br>• Exacerbation<br>• Steroid Dosage	• Blood Eosinophils<br>• FEV1	• Not Stated	• Not Reported	Moderate

Risk of bias for each study assessed using the CASP tool. Grade analysis automatically assumes outcome from observational trial are of low certainty. Data derived from published data and personal communication with authors. FEV1 (forced expiratory volume in one Second), FeNO (fractional exhaled nitric oxide), ACT (Asthma Control Test), ACQ (Asthma Control Questionnaire), SD (standard deviation).

Supplementary Table 4: Study Characteristics of Studies used in Analysis of Dupilumab

Author, Year	N	Age Range	Population	Intervention	Time	Key Outcomes Assessed	Key Biomarkers Assessed	Exacerbation Definition	Adverse Events	Risk of Bias
Dupin, 2020 (39)	62	Median 51 (IQR 44-61)	• Severe Asthma Severe Asthma (non-eosinophilic, non-atopic)	Dupilumab	52 weeks	• Asthma Control: ACT<br>• Exacerbation<br>• Steroid Dosage	• FEV1	• Not Stated	• Adverse Events Reported in 28% of patients	Moderate

Risk of bias for each study assessed using the CASP tool. Grade analysis automatically assumes outcome from observational trial are of low certainty. Data derived from published data and personal communication with authors. FEV1 (forced expiratory volume in one Second), FeNO (fractional exhaled nitric oxide), ACT (Asthma Control Test), ACQ (Asthma Control Questionnaire), SD (standard deviation).

Supplementary Table 5: Grade Assessment for Benralizumab Outcomes

Outcome	ROB	Imprecision	Inconsistency	Indirectness	Publication Bias	Size of Effect	Dose Response	Confounding change effect	Overall
**Δ Annualised Rate of Asthma Exacerbation**	Not Serious (0)<br>3 Studies<br>- 2 Moderate<br>- 1 Low	Not Serious (0)<br>- Small CI<br>- Good magnitude	Not Serious (0)<br>- Point Estimate Similar<br>- Overlapping CI<br>- Consistent Direction of Effect<br>- I^2 = Minimal	Not Serious (0)<br>- Applicable Population<br>- Applicable Intervention<br>- Not surrogate outcome<br>- Sufficient Timeframe	Serious (-1)<br>- Funnel Plot Asymmetrical<br>- Systematic Search performed<br>- Grey Literature Search	Very Large Effect (+2)	N/A	None	Moderate
**Δ ACT Score**	Not Serious (0)<br>4 Studies<br>- 4 Moderate	Not Serious (0)<br>- Wide Size CI<br>- Good magnitude	Not Serious (0)<br>- Point Estimate Similar<br>- Overlapping CI<br>- Consistent Direction of Effect<br>- I^2 = Substantial	Not Serious (0)<br>- Applicable Population<br>- Applicable Intervention<br>- Not surrogate outcome<br>- Sufficient Timeframe	Serious (-1)<br>- Funnel Plot Asymmetrical<br>- Systematic Search performed<br>- Grey Literature Search	Effect Not Large (0)	N/A	None	Very Low
**Δ ACQ-6 Score**	Not Serious (0)<br>2 Studies<br>- 2 Low	Not Serious (0)<br>- Wide Size CI<br>- Good magnitude	Not Serious (0)<br>- Point Estimate Similar<br>- Overlapping CI<br>- Consistent Direction of Effect<br>I^2 = Substantial	Not Serious (0)<br>- Applicable Population<br>- Applicable Intervention<br>- Not surrogate outcome<br>Sufficient Timeframe	Serious (-1)<br>- Funnel Plot Asymmetrical<br>- Systematic Search performed<br>Grey Literature Search	Effect Not Large (0)	N/A	None	Very Low
**Δ Oral Steroid Dose**	Not Serious (0)<br>4 Studies<br>- 3 Moderate<br>- 1 Low	Not Serious (0)<br>- Wide CI<br>- Good magnitude	Serious (-1)<br>- Point Estimate Similar<br>- Overlapping CI<br>- Consistent Direction of Effect<br>- I^2 = Considerable	Not Serious (0)<br>- Applicable Population<br>- Applicable Intervention<br>- Not surrogate outcome<br>- Sufficient Timeframe	Serious (-1)<br>- Funnel Plot Asymmetrical<br>- Systematic Search performed<br>- Grey Literature Search	Effect Not Large (0)	N/A	None	Very Low
**Δ FEV1**	Not Serious (0)<br>5 Studies<br>- 4 Moderate<br>- 1 Low	Not Serious (0)<br>- Moderate Size CI<br>- Good magnitude	Not Serious (0)<br>- Point Estimate Similar<br>- Overlapping CI<br>- Consistent Direction of Effect<br>- I^2 = Substantial	Not Serious (0)<br>- Applicable Population<br>- Applicable Intervention<br>- Not surrogate outcome<br>- Sufficient Timeframe	Not Serious (0)<br>- Funnel Plot Asymmetrical<br>- Systematic Search performed<br>- Grey Literature Search	Effect Not Large (0)	N/A	None	Low
**Δ FeNO**	Not Serious (0)<br>3 Studies<br>- 2 Moderate<br>- 1 Low	Not Serious (0)<br>- Wide Size CI<br>- Good magnitude	Serious (-1)<br>- Point Estimate Similar<br>- Overlapping CI<br>- Consistent Direction of Effect<br>I^2 = Considerable	Not Serious (0)<br>- Applicable Population<br>- Applicable Intervention<br>- Not surrogate outcome<br>- Sufficient Timeframe	Not Serious (0)<br>- Funnel Plot Reasonable Symmetrical<br>- Systematic Search performed<br>- Grey Literature Search	Large Effect (+1)	N/A	None	Low
**Δ Blood Eosinophils**	Not Serious (0)<br>5 Studies<br>- 4 Moderate<br>- 1 Low	Not Serious (0)<br>- Wide CI<br>- Good magnitude	Serious (-1)<br>- Point Estimate Not Similar<br>- Overlapping CI<br>- Consistent Direction of Effect<br>I^2 = Considerable	Not Serious (0)<br>- Applicable Population<br>- Applicable Intervention<br>- Not surrogate outcome<br>- Sufficient Timeframe	Serious (-1)<br>- Funnel Plot Asymmetrical<br>- Systematic Search performed<br>- Grey Literature Search	Effect Not Large (0)	N/A	None	Very Low

FEV1 (forced expiratory volume in one Second), FeNO (fractional exhaled nitric oxide), ACT (Asthma Control Test), ACQ (Asthma Control Questionnaire), CI (Confidence Interval).

Supplementary Table 6: Grade Assessment for Mepolizumab Outcomes

Outcome	ROB	Imprecision	Inconsistency	Indirectness	Publication Bias	Size of Effect	Dose Response	Confounding change effect	Overall
**Δ Annualised Rate of Asthma Exacerbation**	Not Serious (0)<br>7 Studies<br>- 4 Moderate<br>- 3 Low	Not Serious (0)<br>- Small CI<br>- Good magnitude	Not Serious (0)<br>- Point Estimate Similar<br>- Overlapping CI<br>- Consistent Direction of Effect<br>- I^2 = Substantial	Not Serious (0)<br>- Applicable Population<br>- Applicable Intervention<br>- Not surrogate outcome<br>- Sufficient Timeframe	Serious (-1)<br>- Funnel Plot Asymmetrical<br>- Systematic Search performed<br>- Grey Literature Search	Very Large Effect (+2)	N/A	None	Moderate
**Δ ACT Score**	Not Serious (0)<br>8 Studies<br>- 7 Moderate<br>- 1 Low	Not Serious (0)<br>- Moderate Size CI<br>- Good magnitude	Not Serious (0)<br>- Point Estimate Similar<br>- Overlapping CI<br>- Consistent Direction of Effect<br>- I^2 = Substantial	Not Serious (0)<br>- Applicable Population<br>- Applicable Intervention<br>- Not surrogate outcome<br>- Sufficient Timeframe	Serious (-1)<br>- Funnel Plot Asymmetrical<br>- Systematic Search performed<br>- Grey Literature Search	Large Effect (+1)	N/A	None	Low
**Δ ACQ-6 Score**	Not Serious (0)<br>2 Studies<br>- 2 Low	Not Serious (0)<br>- Wide Size CI<br>- Reasonable magnitude	Not Serious (0)<br>- Point Estimate not Similar<br>- Overlapping CI<br>- Consistent Direction of Effect<br>- I^2 = Minimal	Not Serious (0)<br>- Applicable Population<br>- Applicable Intervention<br>- Not surrogate outcome<br>- Sufficient Timeframe	Not Serious (0)<br>- Funnel Plot Reasonable Symmetrical<br>- Systematic Search performed<br>- Grey Literature Search	Effect Not Large (0)	N/A	None	Low
**Δ Oral Steroid Dose**	Not Serious (0)<br>6 Studies<br>- 5 Moderate<br>- 1 Low	Not Serious (0)<br>- Wide CI<br>- Good magnitude	Not Serious (0)<br>- Point Estimate Similar<br>- Overlapping CI<br>- Consistent Direction of Effect<br>- I^2 = Substantial	Not Serious (0)<br>- Applicable Population<br>- Applicable Intervention<br>- Not surrogate outcome<br>- Sufficient Timeframe	Serious (-1)<br>- Funnel Plot asymmetrical<br>- Systematic Search performed<br>- Grey Literature Search	Effect Not Large (0)	N/A	None	Very Low
**Δ FEV1**	Not Serious (0)<br>7 Studies<br>- 6 Moderate<br>- 1 Low	Not Serious (0)<br>- Moderate Size CI<br>- Good magnitude	Not Serious (0)<br>- Point Estimate Similar<br>- Overlapping CI<br>- Consistent Direction of Effect<br>- I^2 = Minimal	Not Serious (0)<br>- Applicable Population<br>- Applicable Intervention<br>- Not surrogate outcome<br>- Sufficient Timeframe	Not Serious (0)<br>- Funnel Plot Symmetrical<br>- Systematic Search performed<br>- Grey Literature Search	Effect Not Large (0)	N/A	None	Low
**Δ FeNO**	Not Serious (0)<br>7 Studies<br>- 5 Moderate<br>- 2 Low	Not Serious (0)<br>- Moderate Size CI<br>- Good magnitude	Not Serious (0)<br>- Point Estimate Similar<br>- Overlapping CI<br>- Consistent Direction of Effect<br>I^2 = Minimal	Not Serious (0)<br>- Applicable Population<br>- Applicable Intervention<br>- Not surrogate outcome<br>- Sufficient Timeframe	Not Serious (0)<br>- Funnel Plot Reasonable Symmetrical<br>- Systematic Search performed<br>- Grey Literature Search	Large Effect (+1)	N/A	None	Moderate
**Δ Blood Eosinophils**	Not Serious (0)<br>8 Studies<br>- 6 Moderate<br>- 2 Low	Not Serious (0)<br>- Wide CI<br>- Good magnitude	Serious (-1)<br>- Point Estimate Not Similar<br>- Overlapping CI<br>- Consistent Direction of Effect<br>I^2 = Considerable	Not Serious (0)<br>- Applicable Population<br>- Applicable Intervention<br>- Not surrogate outcome<br>- Sufficient Timeframe	Serious (-1)<br>- Funnel Plot Asymmetrical<br>- Systematic Search performed<br>- Grey Literature Search	Effect Not Large (0)	N/A	None	Very Low

FEV1 (forced expiratory volume in one Second), FeNO (fractional exhaled nitric oxide), ACT (Asthma Control Test), CI (Confidence Interval).

Supplementary Table 7: Grade Assessment for Reslizumab Outcomes

Outcome	ROB	Imprecision	Inconsistency	Indirectness	Publication Bias	Size of Effect	Dose Response	Confounding change effect	Overall
**Δ Annualised Rate of Asthma Exacerbation**	Not Serious (0)<br>2 Studies<br>- 2 Moderate	Not Serious (0)<br>- Moderate Size<br>- Good magnitude	Not Serious (0)<br>- Point Estimate Similar<br>- Overlapping CI<br>- Consistent Direction of Effect<br>- I^2 = Minimal	Not Serious (0)<br>- Applicable Population<br>- Applicable Intervention<br>- Not surrogate outcome<br>- Sufficient Timeframe	Serious (-1)<br>- Funnel Plot Reasonably symmetrical<br>- Systematic Search performed<br>- Grey Literature Search	Very Large Effect (+2)	N/A	None	Moderate
**Δ Oral Steroid Dose**	Not Serious (0)<br>2 Studies<br>- 2 Moderate	Not Serious (0)<br>- Moderate Size Good magnitude	Not Serious (0)<br>- Point Estimate Similar<br>- Overlapping CI<br>- Consistent Direction of Effect<br>- I^2 = Minimal	Not Serious (0)<br>- Applicable Population<br>- Applicable Intervention<br>- Not surrogate outcome<br>- Sufficient Timeframe	Not Serious (0)<br>- Funnel Plot Reasonably symmetrical<br>- Systematic Search performed<br>- Grey Literature Search	Effect Not Large (0)	N/A	None	Low
**Δ Blood Eosinophils**	Not Serious (0)<br>2 Studies<br>- 2 Moderate	Not Serious (0)<br>- Moderate Size<br>- Good magnitude	Serious (-1)<br>- Point Estimate Not Similar<br>- Overlapping CI<br>- Consistent Direction of Effect<br>- I^2 = Moderate	Not Serious (0)<br>- Applicable Population<br>- Applicable Intervention<br>- Not surrogate outcome<br>- Sufficient Timeframe	Not Serious (0)<br>- Funnel Plot Reasonably symmetrical<br>- Systematic Search performed<br>- Grey Literature Search	Effect Not Large (0)	N/A	None	Very Low

CI (Confidence Interval).

Supplementary Table 8: Risk of Bias Assessment for Identified Studies

Name of author	Was the cohort recruited in an acceptable way?	Was the exposure accurately measured to minimise bias?	Was the outcome accurately measured to minimise bias?	Have the authors identified all important confounding factors?	Have they taken account of the confounding factors in the design and/or analysis?	Was the follow up of subjects complete enough?	Was the follow up of subjects long enough?	Precision	Overall ROB
**Bagnasco**	✓	✓	✓	?	?	✓	?	✓	Moderate
**Bagnasco**	✓	✓	?	✓	✓	✓	✓	✓	Moderate
**Cameli**	✓	✓	✓	?	?	✓	?	✓	Moderate
**Caminati**	✓	✓	✓	?	?	✓	?	✓	Moderate
**Farah**	✓	✓	✓	?	?	✓	?	✓	Moderate
**Ibrahim**	✓	✓	✓	X	?	✓	✓	✓	Moderate
**Kallieri**	✓	✓	?	?	X	✓	✓	✓	Moderate
**Kavanagh**	✓	✓	✓	✓	✓	✓	✓	✓	Low
**Kotisalmi**	✓	✓	✓	✓	?	✓	✓	X	Moderate
**Numata**	✓	✓	?	✓	✓	✓	✓	X	Moderate
**Numata**	✓	✓	✓	✓	✓	✓	?	X	Moderate
**Numata**	✓	✓	✓	✓	?	✓	?	X	Moderate
**Padilla-Galo**	✓	✓	✓	✓	?	✓	?	✓	Moderate
**Pelaia**	✓	✓	✓	✓	✓	✓	?	X	Moderate
**Pelaia**	✓	✓	✓	✓	?	✓	✓	✓	Moderate
**Schleich**	?	?	✓	?	?	✓	✓	✓	High
**Sposatoa**	✓	✓	✓	✓	✓	✓	✓	✓	Low
**Strauss**	✓	✓	✓	✓	?	✓	✓	✓	Moderate
**Van Toor**	✓	✓	✓	✓	✓	✓	✓	✓	Low
**Kavanagh**	✓	✓	✓	✓	✓	✓	✓	✓	Low

✓ - Yes, X - No, ? – Unclear.

Supplementary Table 9: Exploration of Heterogeneity by Meta regression

Variable	Drug	Heterogeneity	Co-efficient (SE)	P-Value	95% Confidence Interval
Exacerbation Rate	Mepolizumab	83.62%	-0.0017423 (+/- 0.0009)	0.063	-0.004 to 0.0000*
	Benralizumab	27.97%	-0.00133 (+/- 0.0009)	0.155	-0.003 to -0.0005*
	Reslizumab	0.00%	-0.0013333 (+/- 0.0152)	0.930	-0.0284 to -0.0310*
FEV1	Mepolizumab	12.20%	-0.00003 (+/-0.0001)	0.828	-0.0003 to 0.0003*
	Benralizumab	84.76%	0.0006703 (+/- 0.0002)	**0.003**	0.0002 to 0.00111*
FENO	Mepolizumab	16.41%	-0.0234 (+/- 0.012)	**0.046**	-0.046 to -0.0039
	Benralizumab	95.23%	-0.0610104 (+/- 0.5212)	0.242	-0.1631 to 0.04114*
Control (ACT)	Mepolizumab	79.50%	0.0016 (+/- 0.003)	0.544	-0.0035 to 0.0067*
	Benralizumab	88.39%	0.0106 (+/- 0.003)	**0.000**	0.0051 to 0.0159*
Control (ACQ-6)	Mepolizumab	0.00%	-0.0017 (+/- 0.007)	0.808	-0.015 to 0.012*
Eosinophil Reduction	Mepolizumab	91.73%	-0.815 (+/- 0.09)	**0.000**	-1.00 to -0.640
	Benralizumab	96.12%	-1.136 (+/- 0.113)	**0.000**	-1.356 to -0.916*
	Reslizumab	44.00%	-0.72727 (+/-1.5790)	0.645	-3.4802 to 1.8250
Steroid Dosage	Mepolizumab	87.30%	-0.00045 (+/- 0.004)	0.909	-0.008 to 0.007*
	Benralizumab	94.84%	-0.0173 (+/- 0.0114)	0.129	-0.04 to 0.0050*
	Reslizumab	0.00%	-.00281 (+/- 0.0049)	0.567	-0.0125 to 0.0068*

A negative co-efficient indicates a decrease in the asthma output variable equivalent to the co-efficient per unit increase in the eosinophil count. SE (Standard Error), FEV1 (forced expiratory volume in one Second), FeNO (fractional exhaled nitric oxide), ACT (Asthma Control Test), ACQ (Asthma Control Questionnaire).

Supplementary Table 10: Change in Asthma outcome parameter with the Active group in Randomised Control Trial Data

Drug	Paper	Number	Details	FEV1		Asthma Control			Exacerbation	
				Change	CI	Test	Change	SE	Change	SE
Mepolizumab	Chupp, 2017 [41]	269	S/C Mepolizumab every 4 weeks	0.176 L	0.051	-	-	-	2.39	-
	Ortega, 2014[42]	194	S/C Mepolizumab every 4 weeks	0.183 L	0.061	ACQ-5	-0.94	0.07	2.97	-
	Bel, 2014[43]	69	S/C Mepolizumab every 4 weeks	0.111 L	0.11	-	-	-	1.86**	-
Benralizumab	Nair, 2017[44]	73	S/C Benralizumab every 4 week for 3 months then every 8 weeks	0.23 L	0.10**	-	-	-	1.97	-
Reslizumab	Castro, 2011[54]	53	IV infusion of Reslizumab every 4 weeks	0.18 L	0.10	ACQ	-0.853	0.12	-	-
						ACQ-5	-0.787	-	-	-
						ACQ-6	-0.838	-	-	-
	Bjermer, 2016[49]	106	IV infusion of Reslizumab every 4 weeks	0.286 L	0.12	ACQ-7	-0.838	0.05	-	-
	Corren, 2016[50]	396	IV infusion of Reslizumab every 4 weeks	0.255 L	0.05	ACQ-7	-0.844	-	-	-
	Castro, 2015*[46]	245	Study a: IV infusion of Reslizumab every 4 weeks	0.24 L	-	ACQ-7	-0.91	-	1	-
		233	Study b: IV infusion of Reslizumab every 4 weeks	0.20 L	-	ACQ-7	-1.02	-	1.04	-

Studies identified using a recent systematic review (18). Data from published studies plus additional data from authors. Number represents total number cited in study within active group being examined. * Represents two duplicate multicentre, double-blind, parallel-group placebo controlled trials. FEV1 (forced expiratory volume in one Second), L (litres), ACQ (Asthma Control Questionnaire). ** Values estimated from figures.

Supplementary Table 11: Baseline Eosinophil Level

		Pre-Intervention		Post-Intervention	
Author, Year	Drug	Mean	Standard Deviation	Mean	Standard Deviation
Bagnasco, 2020	Benralizumab	581	(SD +/- 556)	24	(SD +/- 48)
Numata, 2020	Benralizumab	458	(SD +/- 338)	0	(SD +/- 0)
Padillo-Gala, 2020	Benralizumab	757.2	(SD +/- 278)	15.2	(SD +/- 13.6)
Kavanagh, 2020	Benralizumab	290	(SD +/- 250)	70	(SD +/- 28)
Cameli, 2020	Mepolizumab	905.4	(SD +/- 628.7)	75	(SD +/- 54.8)
Caminati, 2019	Mepolizumab	983	(SD +/- 1021.3)	130.5	(SD +/- 192.3)
Kallieri, 2020	Mepolizumab	703	(SD +/- 537)	661	(SD +/- 456)
Kavanagh, 2020	Mepolizumab	290	(SD +/- 310)	70	(SD +/- 70)
Kotisalmi, 2020	Mepolizumab, Benralizumab, Reslizumab	450	(SD +/- 370)	-	-
Numata, 2020	Mepolizumab	1228	(SD +/- 2360)	99	(SD +/- 131)
Numata, 2019	Mepolizumab	873	(SD +/- 1851)	71	(SD +/- 64)
Pelaia, 2020	Benralizumab	947.9	(SD +/- 602.5)	0.00	(SD +/- 0.00)
Pelaia, 2020	Mepolizumab	989	(SD +/- 1082)	100.1	(SD +/- 124.2)
Sposato, 2020	Mepolizumab	712	-	-	-
Strauss, 2018	Mepolizumab	438	-	-	-
Van Toor, 2020	Mepolizumab	450	(SD +/- 450)	-	-
Ibrahim, 2019	Reslizumab	780	(SD +/- 510)	40	(SD +/- 30)

SD (Standard Deviation).

Supplementary Table 12: PRISM Checklist

Section/topic	#	Checklist item	Reported on page #
**TITLE**			
Title	1	Identify the report as a systematic review, meta-analysis, or both.	1
**ABSTRACT**			
Structured summary	2	Provide a structured summary including, as applicable: background; objectives; data sources; study eligibility criteria, participants, and interventions; study appraisal and synthesis methods; results; limitations; conclusions and implications of key findings; systematic review registration number.	2
**INTRODUCTION**			
Rationale	3	Describe the rationale for the review in the context of what is already known.	3
Objectives	4	Provide an explicit statement of questions being addressed with reference to participants, interventions, comparisons, outcomes, and study design (PICOS).	4
**METHODS**			
Protocol and registration	5	Indicate if a review protocol exists, if and where it can be accessed (e.g., Web address), and, if available, provide registration information including registration number.	4
Eligibility criteria	6	Specify study characteristics (e.g., PICOS, length of follow-up) and report characteristics (e.g., years considered, language, publication status) used as criteria for eligibility, giving rationale.	4-5
Information sources	7	Describe all information sources (e.g., databases with dates of coverage, contact with study authors to identify additional studies) in the search and date last searched.	4-5
Search	8	Present full electronic search strategy for at least one database, including any limits used, such that it could be repeated.	SM1
Study selection	9	State the process for selecting studies (i.e., screening, eligibility, included in systematic review, and, if applicable, included in the meta-analysis).	5
Data collection process	10	Describe method of data extraction from reports (e.g., piloted forms, independently, in duplicate) and any processes for obtaining and confirming data from investigators.	5
Data items	11	List and define all variables for which data were sought (e.g., PICOS, funding sources) and any assumptions and simplifications made.	4
Risk of bias in individual studies	12	Describe methods used for assessing risk of bias of individual studies (including specification of whether this was done at the study or outcome level), and how this information is to be used in any data synthesis.	5-6
Summary measures	13	State the principal summary measures (e.g., risk ratio, difference in means).	5
Synthesis of results	14	Describe the methods of handling data and combining results of studies, if done, including measures of consistency (e.g., I^2) for each meta-analysis.	5

Section/topic	#	Checklist item	Reported on page #
Risk of bias across studies	15	Specify any assessment of risk of bias that may affect the cumulative evidence (e.g., publication bias, selective reporting within studies).	5-6
Additional analyses	16	Describe methods of additional analyses (e.g., sensitivity or subgroup analyses, meta-regression), if done, indicating which were pre-specified.	5-6
**RESULTS**			
Study selection	17	Give numbers of studies screened, assessed for eligibility, and included in the review, with reasons for exclusions at each stage, ideally with a flow diagram.	5, 15
Study characteristics	18	For each study, present characteristics for which data were extracted (e.g., study size, PICOS, follow-up period) and provide the citations.	18-20
Risk of bias within studies	19	Present data on risk of bias of each study and, if available, any outcome level assessment (see item 12).	21-23
Results of individual studies	20	For all outcomes considered (benefits or harms), present, for each study: (a) simple summary data for each intervention group (b) effect estimates and confidence intervals, ideally with a forest plot.	10, 31-46
Synthesis of results	21	Present results of each meta-analysis done, including confidence intervals and measures of consistency.	10
Risk of bias across studies	22	Present results of any assessment of risk of bias across studies (see Item 15).	16-20
Additional analysis	23	Give results of additional analyses, if done (e.g., sensitivity or subgroup analyses, meta-regression [see Item 16]).	6-11
**DISCUSSION**			
Summary of evidence	24	Summarize the main findings including the strength of evidence for each main outcome; consider their relevance to key groups (e.g., healthcare providers, users, and policy makers).	11
Limitations	25	Discuss limitations at study and outcome level (e.g., risk of bias), and at review-level (e.g., incomplete retrieval of identified research, reporting bias).	12
Conclusions	26	Provide a general interpretation of the results in the context of other evidence, and implications for future research.	13
**FUNDING**			
Funding	27	Describe sources of funding for the systematic review and other support (e.g., supply of data); role of funders for the systematic review.	1-2

From: Moher D, Liberati A, Tetzlaff J, Altman DG, The PRISMA Group (2009). Preferred Reporting Items for Systematic Reviews and Meta-Analyses: The PRISMA Statement. PLoS Med 6(7): e1000097. doi:10.1371/journal.pmed1000097

Supplementary Table 13: Funding and Conflicts of Interest

Author, Year	Drug	Industry Funding/Declared Conflicts of Interest
Bagnasco, 2020 (19)	Benralizumab	None Declared
Numata, 2020 (21)	Benralizumab	None Declared
Padillo-Gala, 2020 (20)	Benralizumab	None Declared
Pelaia, 2020 (24)	Benralizumab	None Declared
Kavanagh, 2020 (22)	Benralizumab	3 authors reported advisory board and speaker fees and congress travel support from GSK, Astrazeneca, Chiesi, Napp, and Teva pharmaceuticals. 1 author reported travel support from Teva. 1 author reports speaker fees and congress travel support from AstraZeneca.
Kotisalmi, 2020 (23)	Benralizumab<br>Mepolizumab<br>Reslizumab	None Declared
Bagnasco, 2019 (25)	Mepolizumab	None Declared
Cameli, 2020 (26)	Mepolizumab	None Declared
Caminati, 2019 (27)	Mepolizumab	None Declared
Farah, 2019 (28)	Mepolizumab	None Declared
Kallieri, 2020 (29)	Mepolizumab	None Declared
Kavanagh, 2020 (30)	Mepolizumab	1 author reported travel support from Teva. 3 authors reported advisory board and speaker fees and congress travel support from GSK, AstraZeneca, Chiesi, Napp, and Teva pharmaceuticals. 1 author reported speaker fees and congress travel support from Astrazeneca.
Kotisalmi, 2020 (23)	Mepolizumab	None Declared
Numata, 2020 (32)	Mepolizumab	None Declared
Numata, 2019 (31)	Mepolizumab	None Declared
Pelaia, 2020 (33)	Mepolizumab	None Declared
Schleich, 2020 (35)	Mepolizumab	None Declared
Sposato, 2020 (34)	Mepolizumab	None Declared
Strauss, 2018 (36)	Mepolizumab	None Declared
Van Toor, 2020 (37)	Mepolizumab	Sponsored by a grant from GlaxoSmithKline
Ibrahim, 2019 (38)	Reslizumab	The drug used in the study was provided as part of an early access program, but the company had no role in the design, collection, analysis of data or preparation of the manuscript.

Supplementary Table 14: Funding and Conflicts of Interest: A Sensitivity Analysis

Variable	Drug	Sensitivity Analysis Outcome	Overall Outcome
Δ Exacerbations	Mepolizumab	-3.43 [-3.69, -3.17]	-3.17 [-3.74, -2.59]
	Benralizumab	-3.94 [-5.66, -2.21]	-3.79 [-4.53, -3.04]
	Reslizumab	N/A	-6.72 [-8.47, -4.97]
Δ Control	Mepolizumab	+6.15 [5.14, 7.15]	+6.15 [5.14, 7.15]
	Benralizumab	+5.82 [3.39, 8.25]	+5.82 [3.39, 8.25]
	Reslizumab	N/A	N/A
Δ FEV1	Mepolizumab	+0.17 [0.10, 0.25]	+0.17 [0.11, 0.24]
	Benralizumab	+0.24 [0.00, 0.48}	+0.21 [0.08, 0.34]
	Reslizumab	N/A	N/A
Δ FeNO	Mepolizumab	-15.43 [-21.27, -9.59]	-14.23 [-19.71, -8.75}
	Benralizumab	-15.28 [-54.90, 24.33]	-14.18 [-36.54, 8.17]
	Reslizumab	N/A	N/A
Δ Eosinophils	Mepolizumab	-669.48 [-825.65, -513.50]	-609.19 [-793.20, -425.18]
	Benralizumab	-608.59 [-852.28, -364.89]	-518.68 [-820.24, -217.12]
	Reslizumab	N/A	-603.60 [-838.69, -368.51]
Δ Steroids	Mepolizumab	-4.85 [-7.30, -2.39]	-5.30 [-7.50, -3.10]
	Benralizumab	-8.08 [-16.20, 0.05]	-8.35 [-13.83, -2.87]
	Reslizumab	N/A	-3.90 [-5.26, -2.54]

Supplementary Table 15: Studies at low risk of bias: a sensitivity analysis

Variable	Drug	Outcome	Overall Outcome
Δ Exacerbations	Mepolizumab	-2.80 [-3.78, -1.82]	-3.17 [-3.74, -2.59]
	Benralizumab	N/A	-3.79 [-4.53, -3.04]
	Reslizumab	N/A	-6.72 [-8.47, -4.97]
Δ Control	Mepolizumab	N/A	6.15 [5.14, 7.15]
	Benralizumab	N/A	5.82 [3.39, 8.25]
	Reslizumab	N/A	N/A
Δ FEV1	Mepolizumab	N/A	+0.17 [0.11, 0.24]
	Benralizumab	N/A	+0.21 [0.08, 0.34]
	Reslizumab	N/A	N/A
Δ FeNO	Mepolizumab	-6.59 [-15.12, 2.13]	-14.23 [-19.71, -8.75}
	Benralizumab	N/A	-14.18 [-36.54, 8.17]
	Reslizumab	N/A	N/A
Δ Eosinophils	Mepolizumab	-379.37 [-690.51, -68.22]	-609.19 [-793.20, -425.18]
	Benralizumab	N/A	-518.68 [-820.24, -217.12]
	Reslizumab	N/A	-603.60 [-838.69, -368.51]
Δ Steroids	Mepolizumab	N/A	-5.30 [-7.50, -3.10]
	Benralizumab	N/A	-8.35 [-13.83, -2.87]
	Reslizumab	N/A	-3.90 [-5.26, -2.54]

Supplementary Table 16: Retrospective and Prospective Anti-IL5 Biologics Studies

Mepolizumab		Benralizumab		Reslizumab	
Retrospective	*Prospective*	*Retrospective*	*Prospective*	*Retrospective*	*Prospective*
Bagnasco, 2019 (25)	Farah, 2019 (28)	Bagnasco, 2020 (19)	N/A	Ibrahim, 2019 (38)	N/A
Cameli, 2020 (26)	Kallieri, 2020 (29)	Numata, 2020 (21)		Kotisalmi, 2020 (23)	
Caminati, 2019 (27)	Schleich, 2020 (35)	Pelaia, 2020 (24)			
Kavanagh, 2020 (30)		Kavanagh, 2020 (22)			
Kotisalmi, 2020 (23)		Kotisalmi, 2020 (23)			
Numata, 2020 (32)		Padillo-Gala, 2020 (20)			
Numata, 2019 (31)					
Pelaia, 2020 (33)					
Sposato, 2020 (34)					
Strauss, 2018 (36)					
Van Toor, 2020 (37)					

Supplementary Table 17: Retrospective Anti-IL5 Biologics Studies: Sensitivity Analysis

Variable	Drug	Retrospective Sub-Group	Overall Outcome
Δ Exacerbations	Mepolizumab	-3.32 [-3.59, -3.05]	-3.17 [-3.74, -2.59]
	Benralizumab	As per main text	-3.79 [-4.53, -3.04]
	Reslizumab	As per main text	-6.72 [-8.47, -4.97]
Δ Control	Mepolizumab	5.94 [4.38,7.50]	+6.15 [5.14, 7.15]
	Benralizumab	As per main text	+5.82 [3.39, 8.25]
	Reslizumab	As per main text	N/A
Δ FEV1	Mepolizumab	As per main text	+0.17 [0.11, 0.24]
	Benralizumab	As per main text	+0.21 [0.08, 0.34]
	Reslizumab	As per main text	N/A
Δ FeNO	Mepolizumab	-14.23 [-19.71, -8.75]	-14.23 [-19.71, -8.75}
	Benralizumab	As per main text	-14.18 [-36.54, 8.17]
	Reslizumab	As per main text	N/A
Δ Eosinophils	Mepolizumab	-645.03 [-898.51, -391.56]	-609.19 [-793.20, -425.18]
	Benralizumab	As per main text	-518.68 [-820.24, -217.12]
	Reslizumab	As per main text	-603.60 [-838.69, -368.51]
Δ Steroid	Mepolizumab	-5.75 [-8.26, -3.23]	-5.30 [-7.50, -3.10]
	Benralizumab	As per main text	-8.35 [-13.83, -2.87]
	Reslizumab	As per main text	-3.90 [-5.26, -2.54]

Supplementary Figure 1: Mepolizumab Annualised Exacerbation Rate Forest Plot

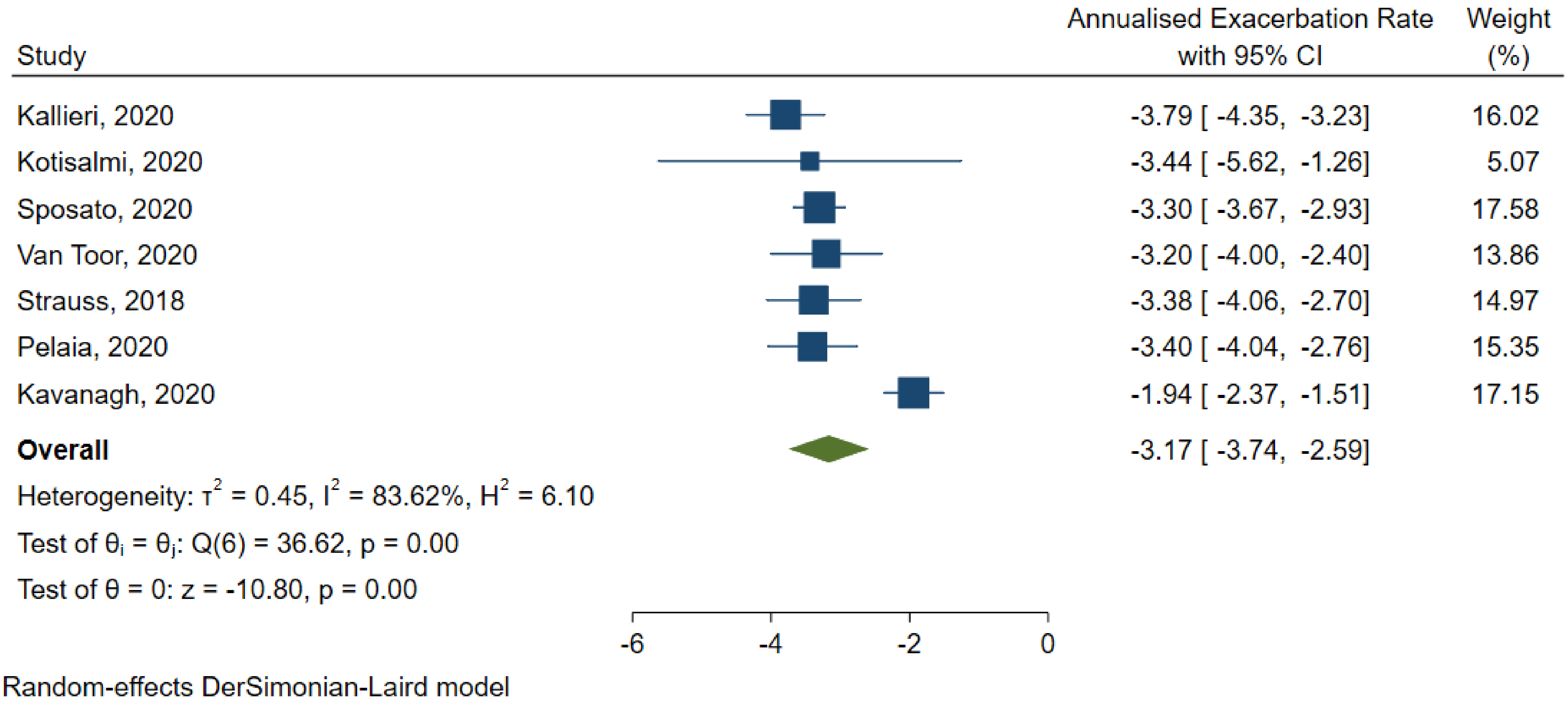

Supplementary Figure 2: Benralizumab Annualised Exacerbation Rate Forest Plot

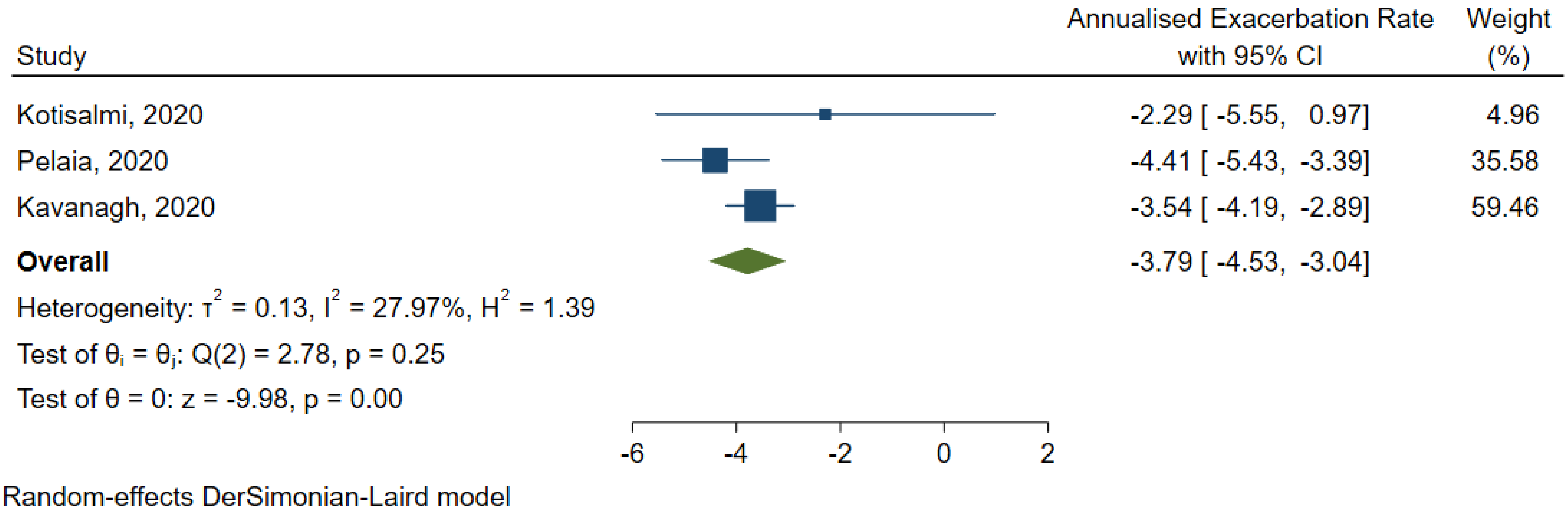

Supplementary Figure 3: Reslizumab Annualised Exacerbation Rate Forest Plot

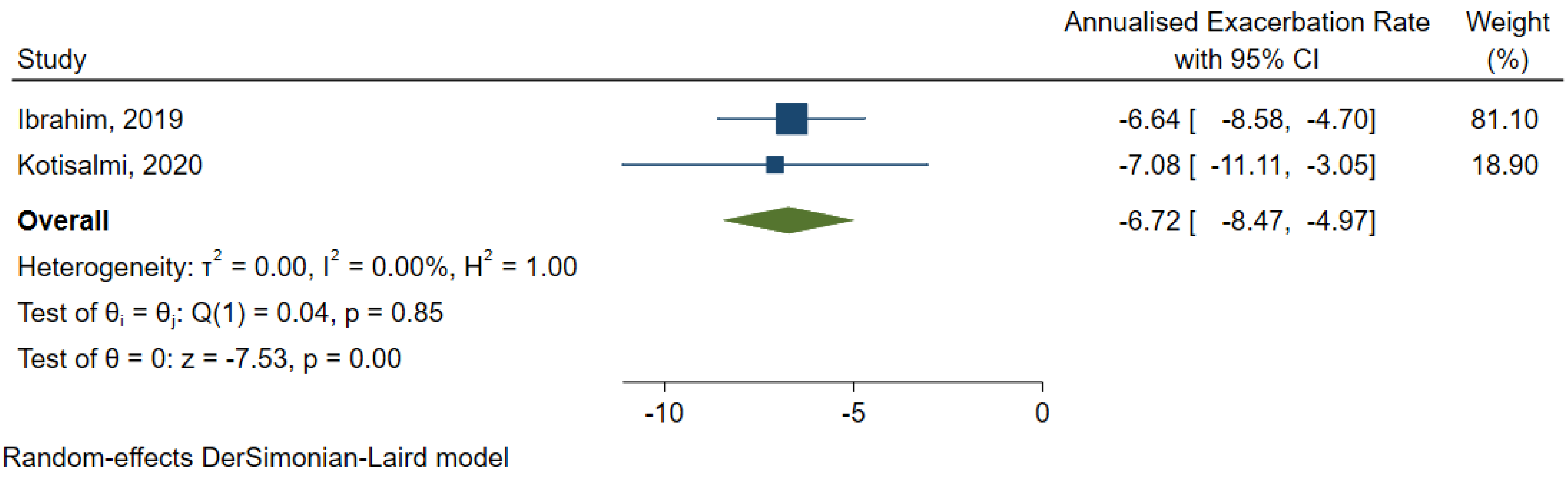

Supplementary Figure 4: Mepolizumab Change in FEV1 Forest Plot

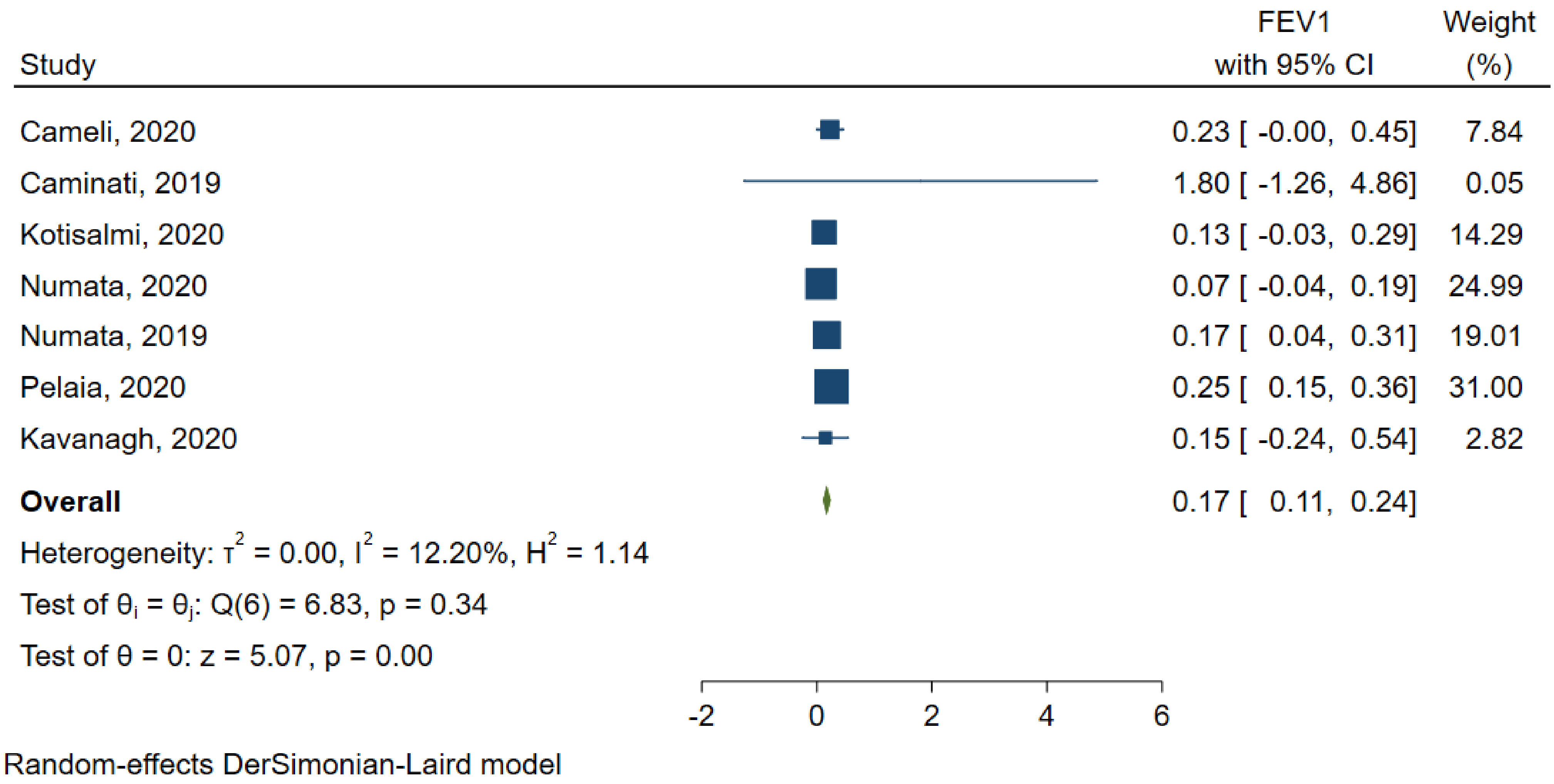

Supplementary Figure 5: Benralizumab Change in FEV1 Forest Plot

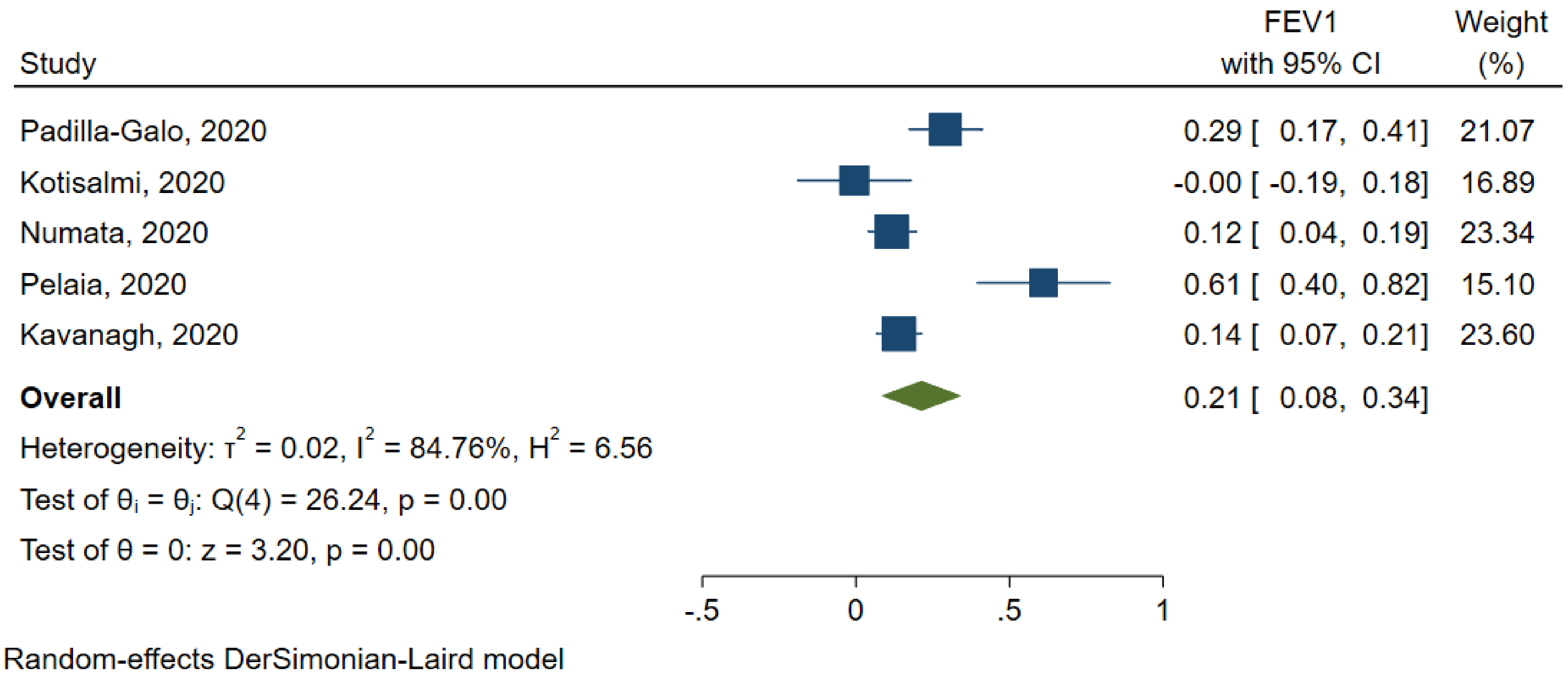

Supplementary Figure 6: Mepolizumab Change in FeNO Forest Plot

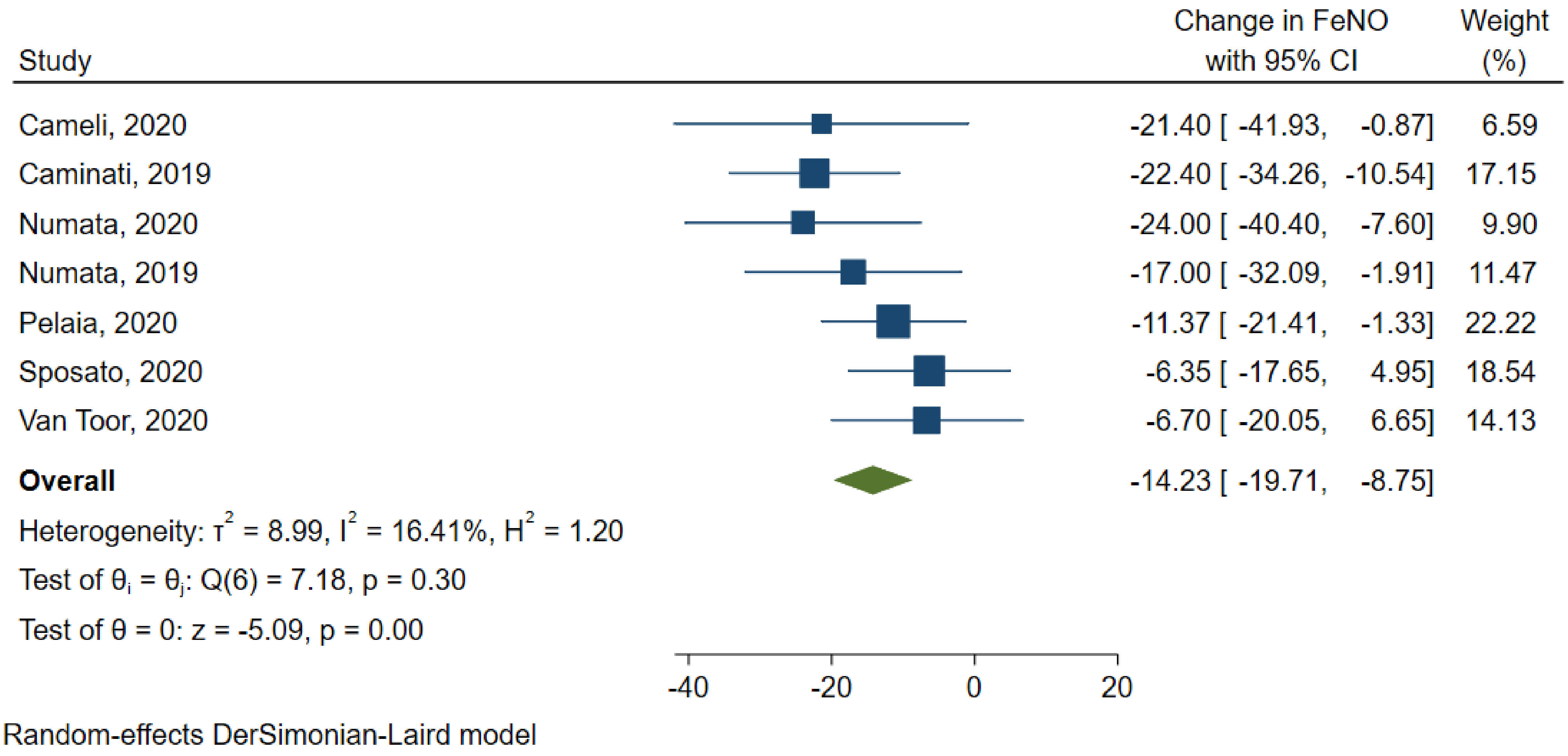

Supplementary Figure 7: Benralizumab Change in FeNO Forest Plot

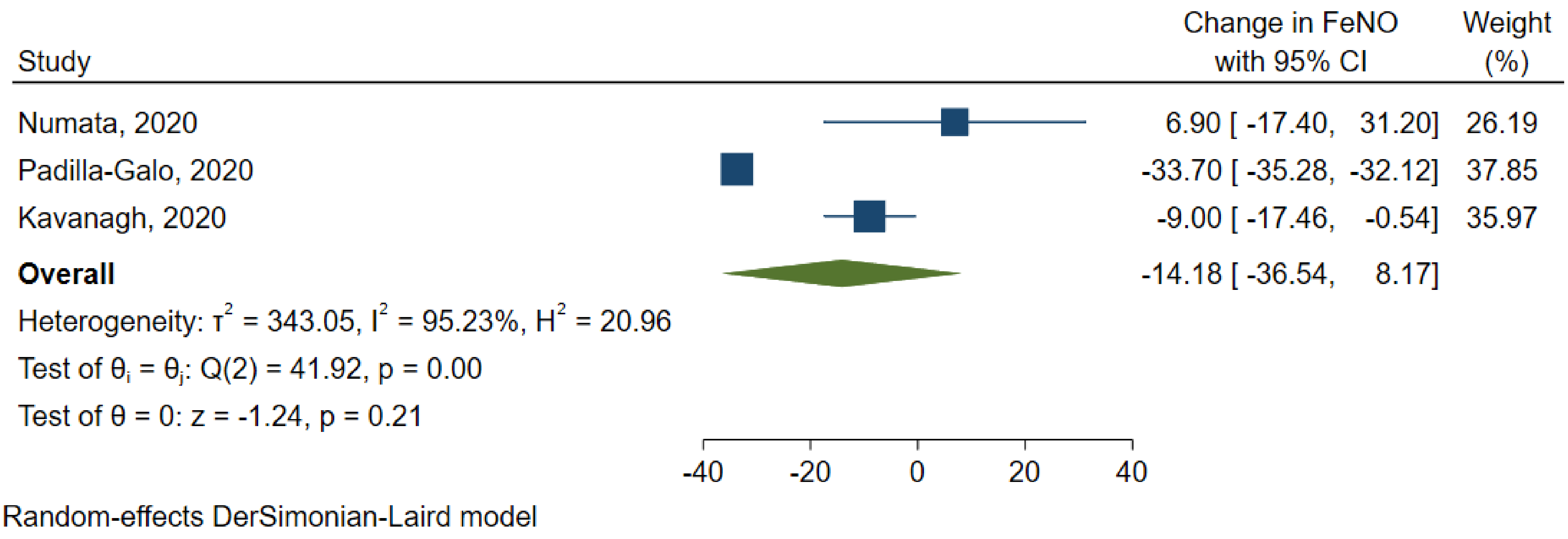

Supplementary Figure 8: Mepolizumab Change in ACT Forest Plot

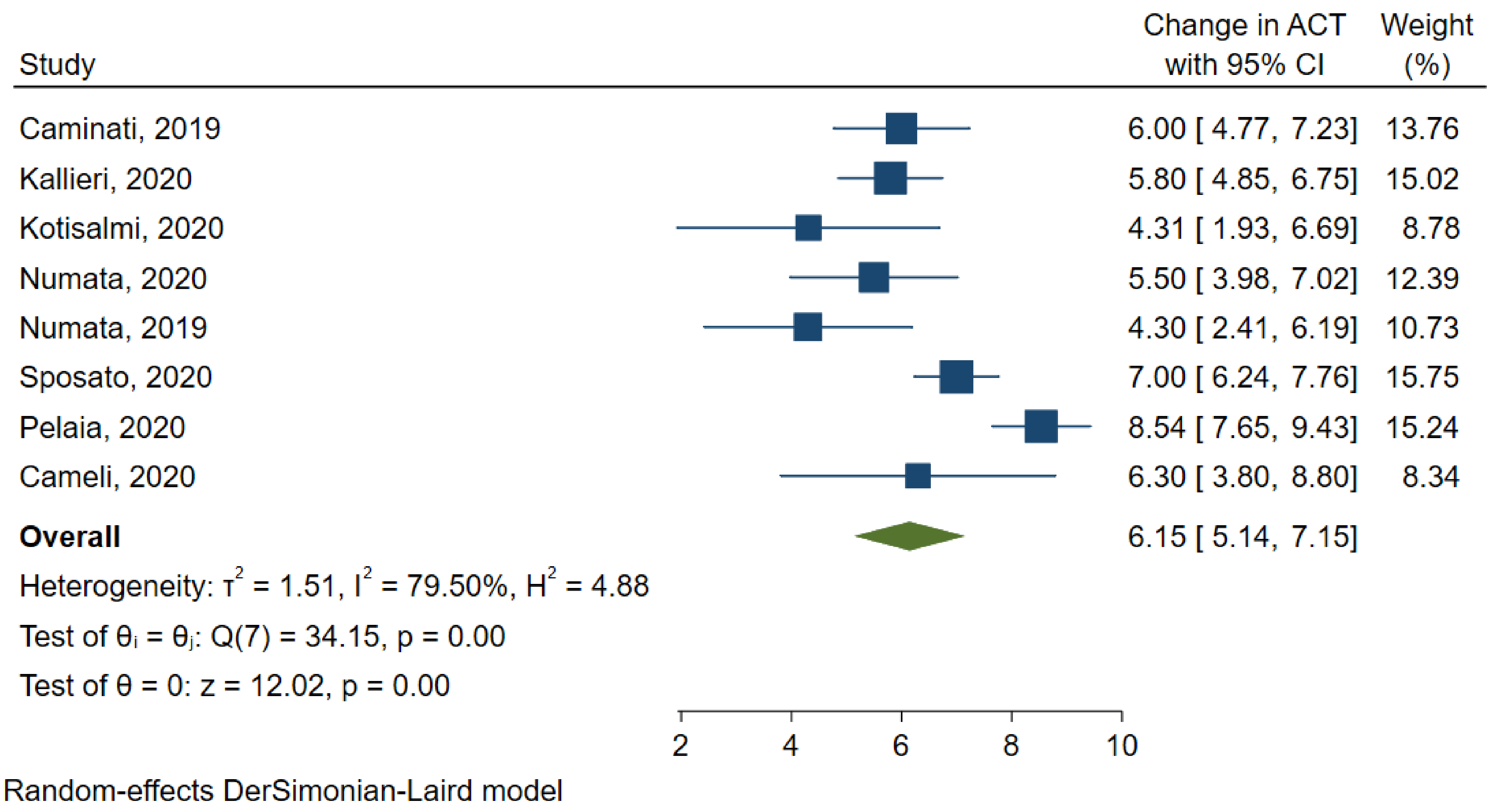

Supplementary Figure 9: Benralizumab Change in ACT Forest Plot

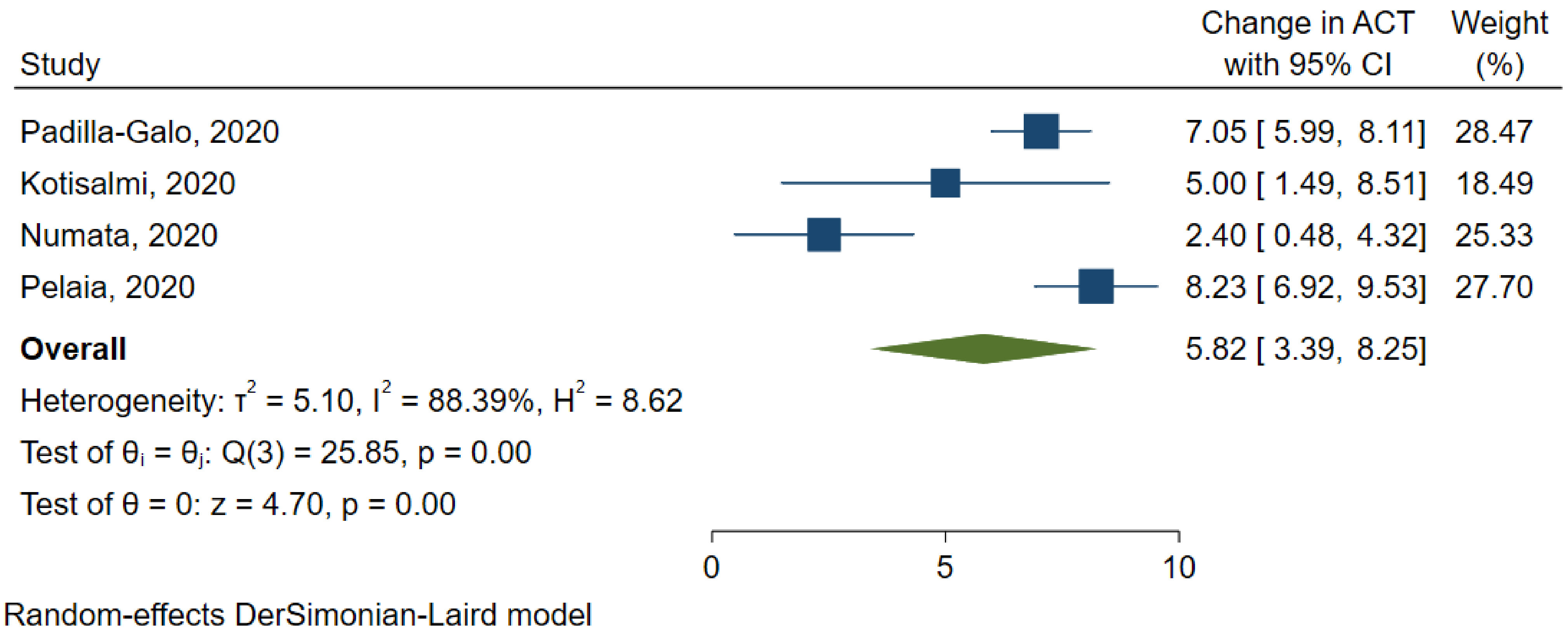

Supplementary Figure 10: Mepolizumab Change in ACQ-6 Forest Plot

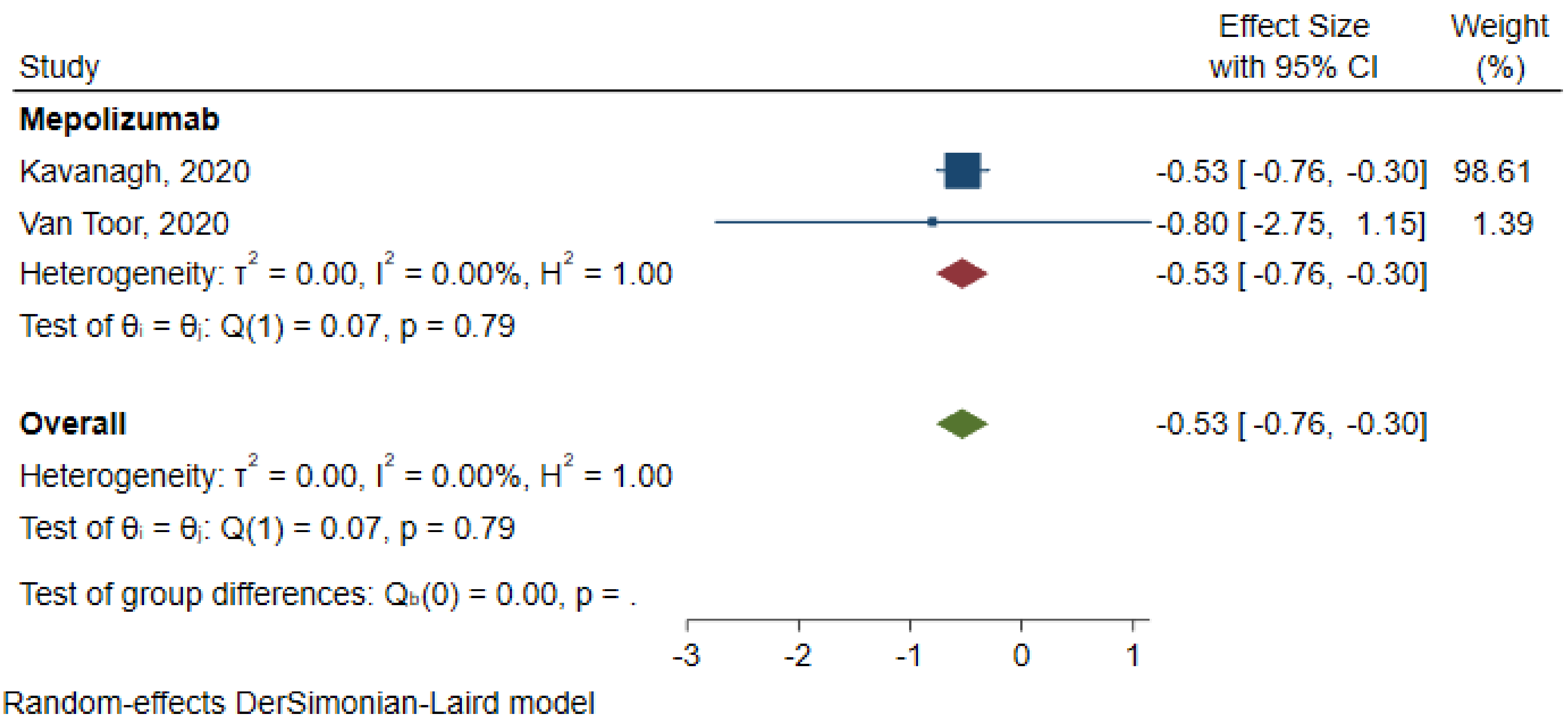

Supplementary Figure 11: Mepolizumab Change in Eosinophils Forest Plot

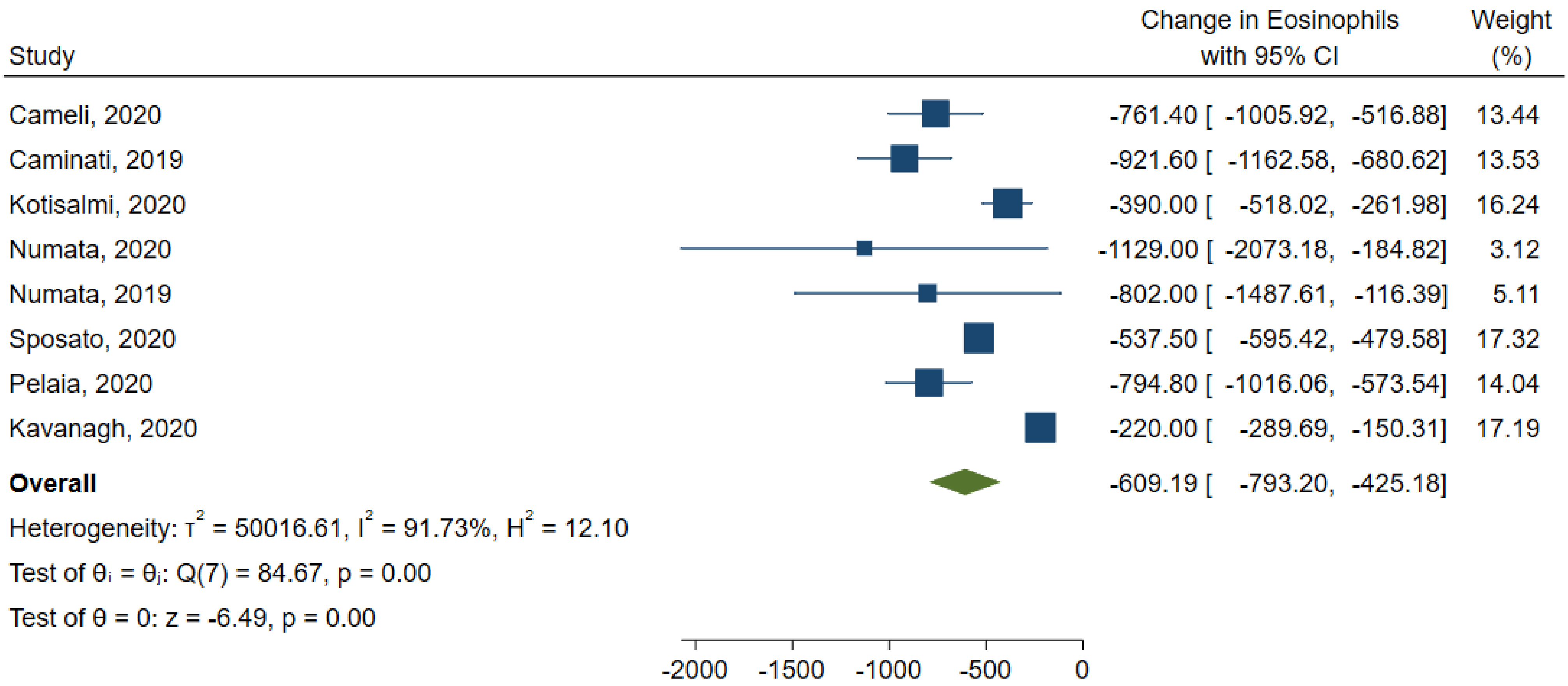

Random-effects DerSimonian-Laird model

Supplementary Figure 12: Benralizumab Change in Eosinophils Forest Plot

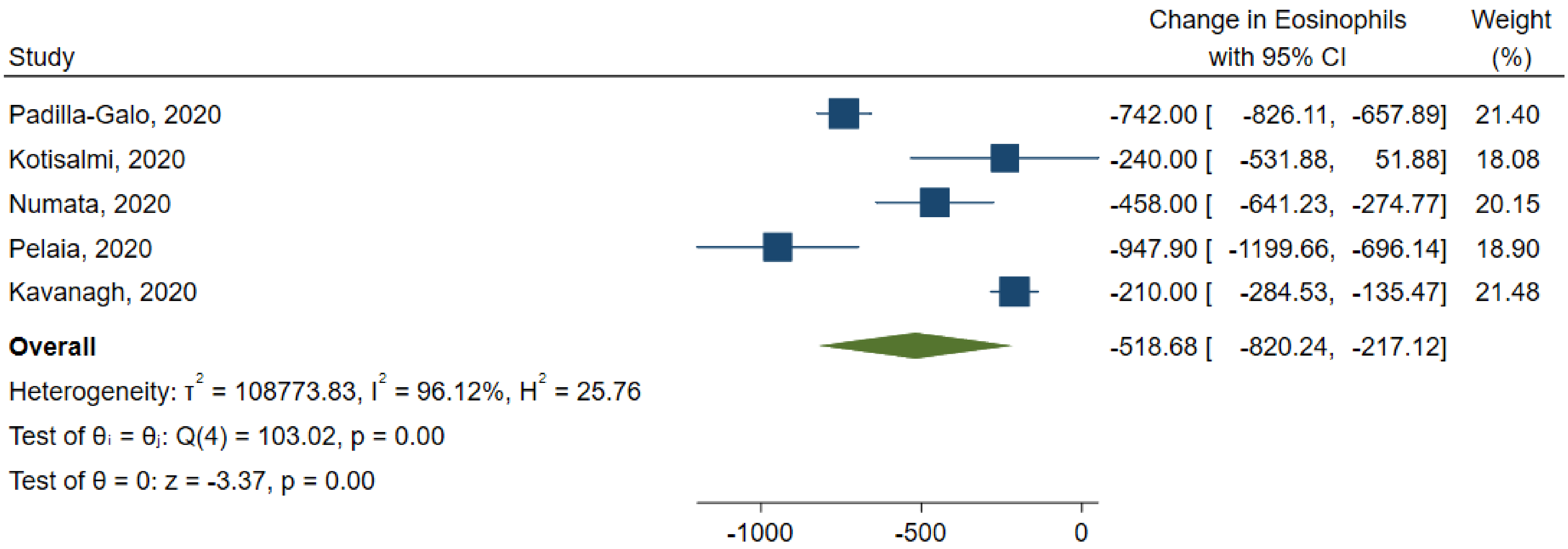

Random-effects DerSimonian-Laird model

Supplementary Figure 13: Reslizumab Change in Eosinophils Forest Plot

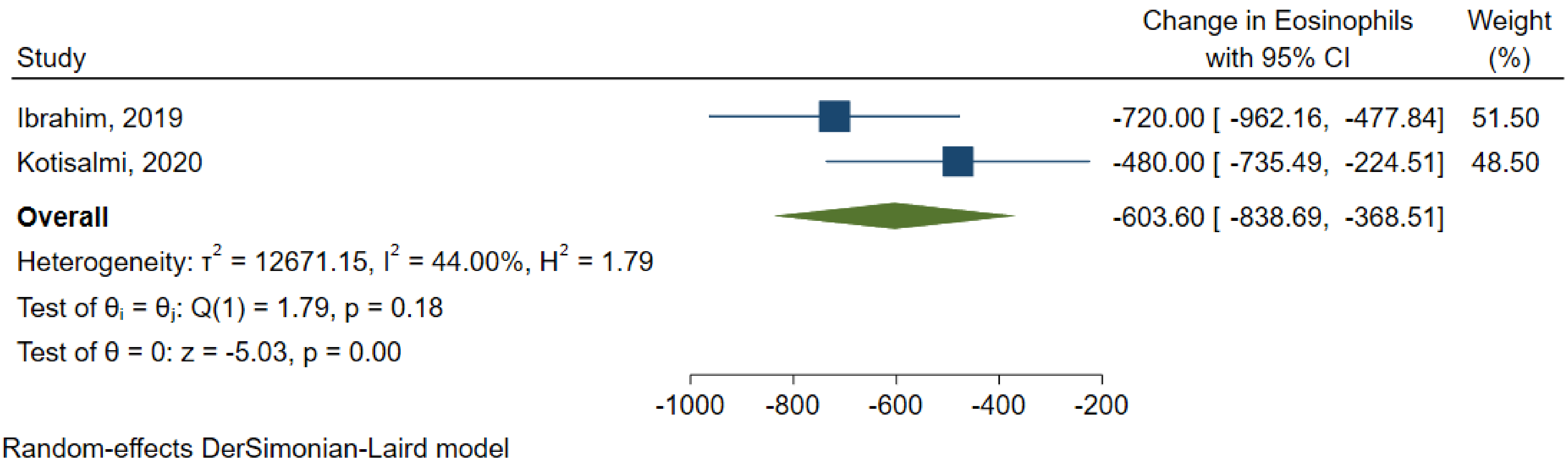

Supplementary Figure 14: Mepolizumab Change in Steroid Dosage Forest Plot

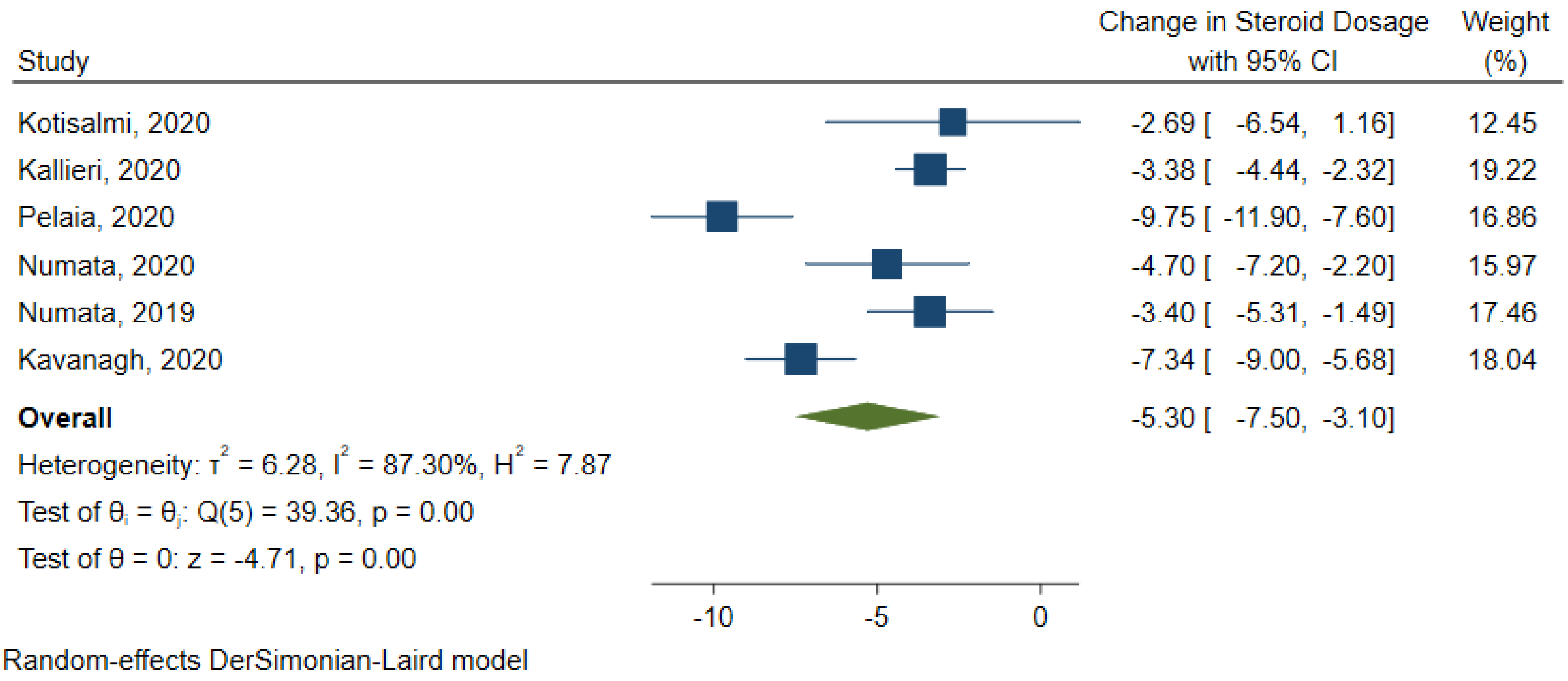

Supplementary Figure 15: Benralizumab Change in Steroid Dosage Forest Plot

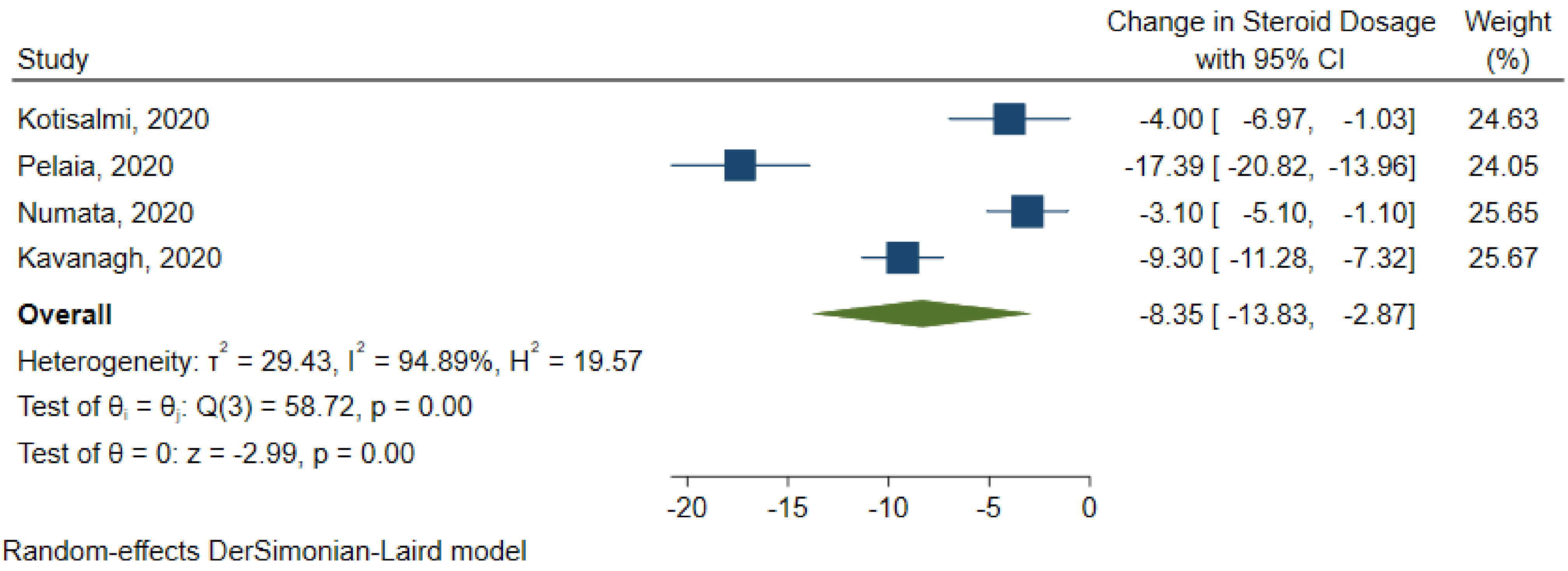

Supplementary Figure 16: Reslizumab Change in Steroid Dosage Forest Plot

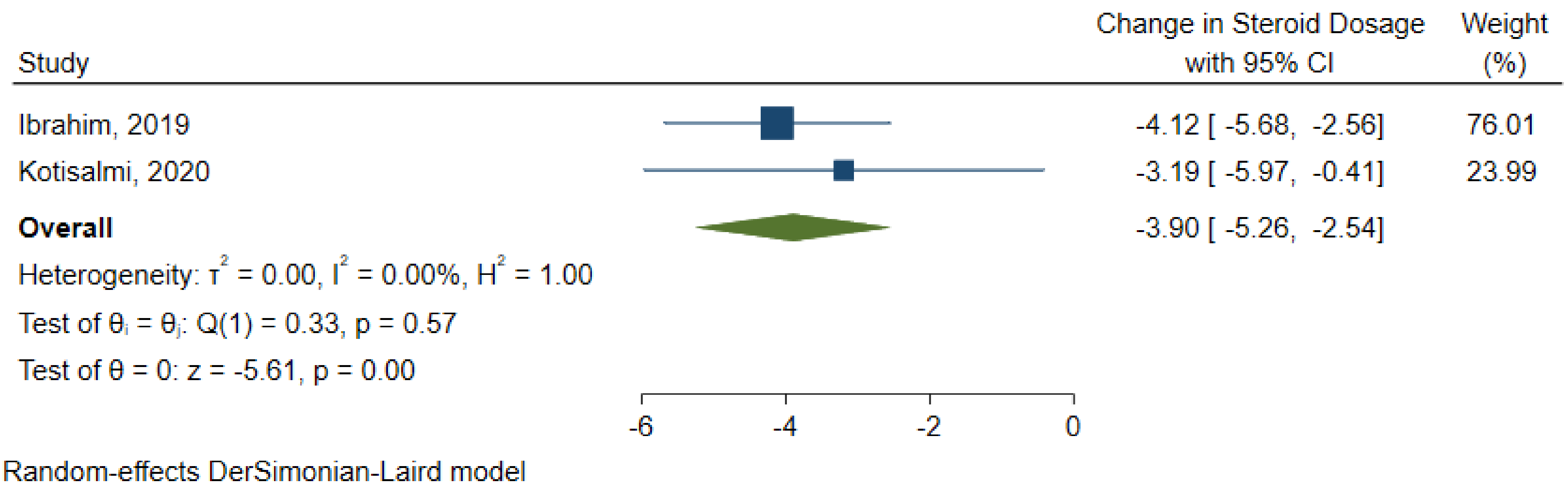

Supplementary Figure 17: Annualised Exacerbation Rate Funnel Plot

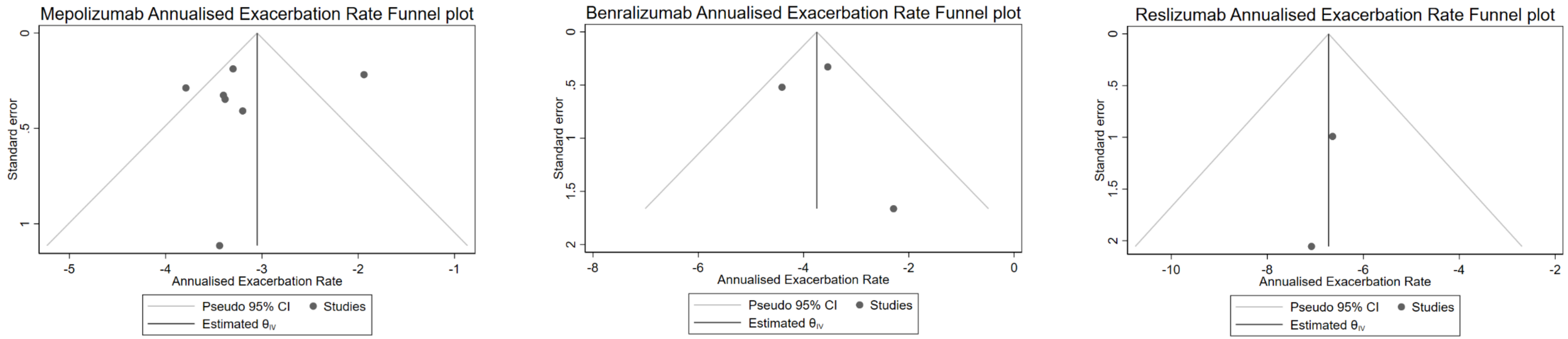

CI (Confidence Interval).

Supplementary Figure 18: FEV1 Change Funnel Plot

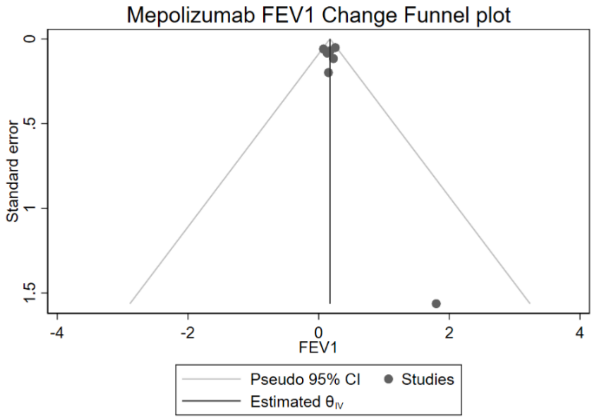

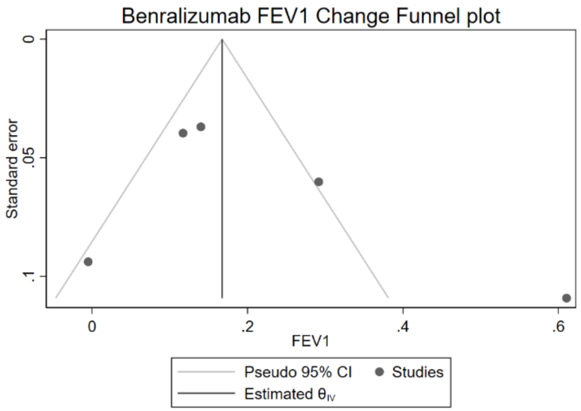

CI (Confidence Interval), FEV1 (forced expiratory volume in one Second).

Supplementary Figure 19: FeNO Change Funnel Plot

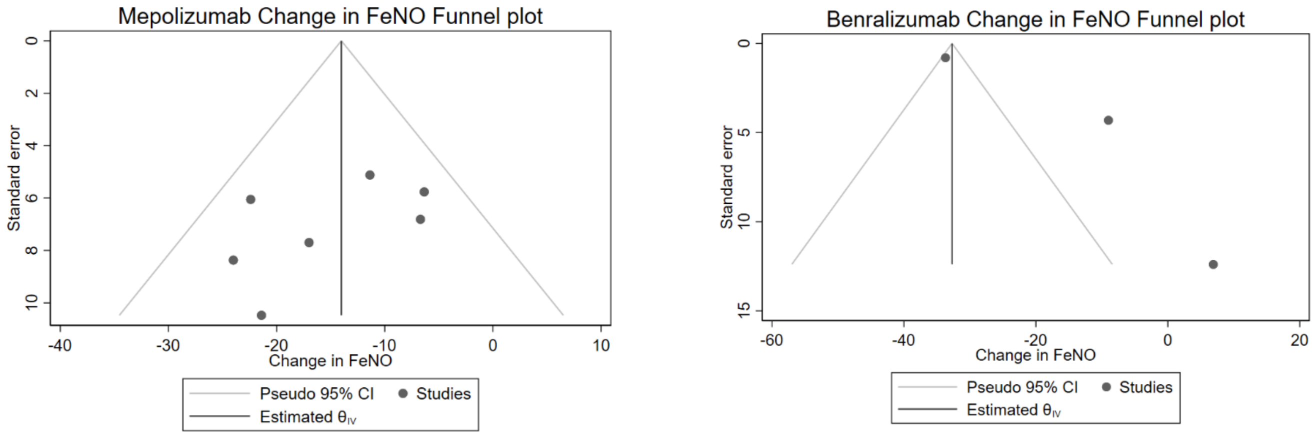

CI (Confidence Interval), FeNO (fractional exhaled nitric oxide).

Supplementary Figure 20: Change in Control Funnel Plot

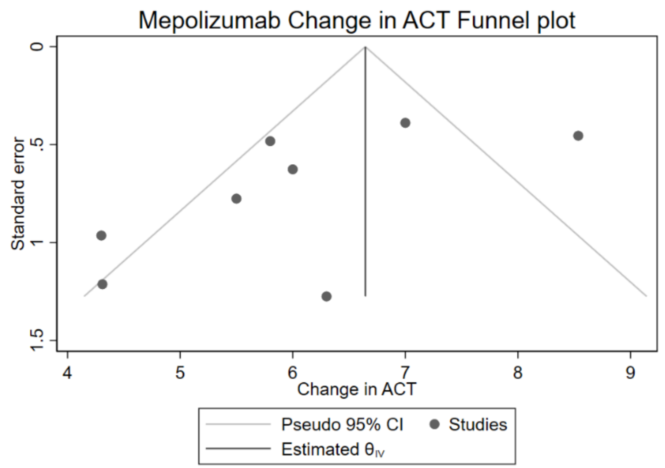

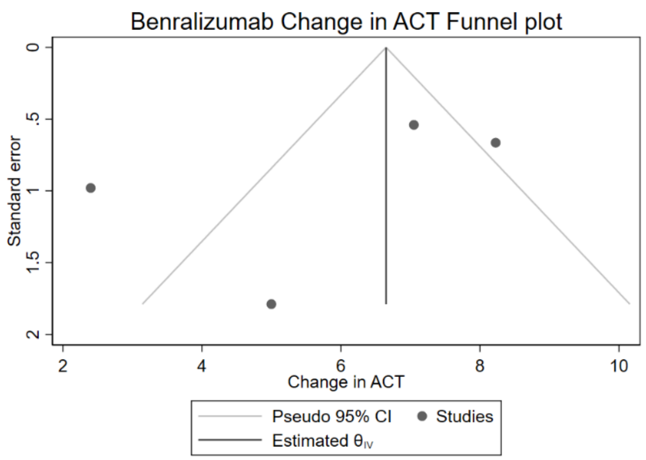

ACT (Asthma Control Test), CI (Confidence Interval).

Supplementary Figure 21: Change in Eosinophils Funnel Plot

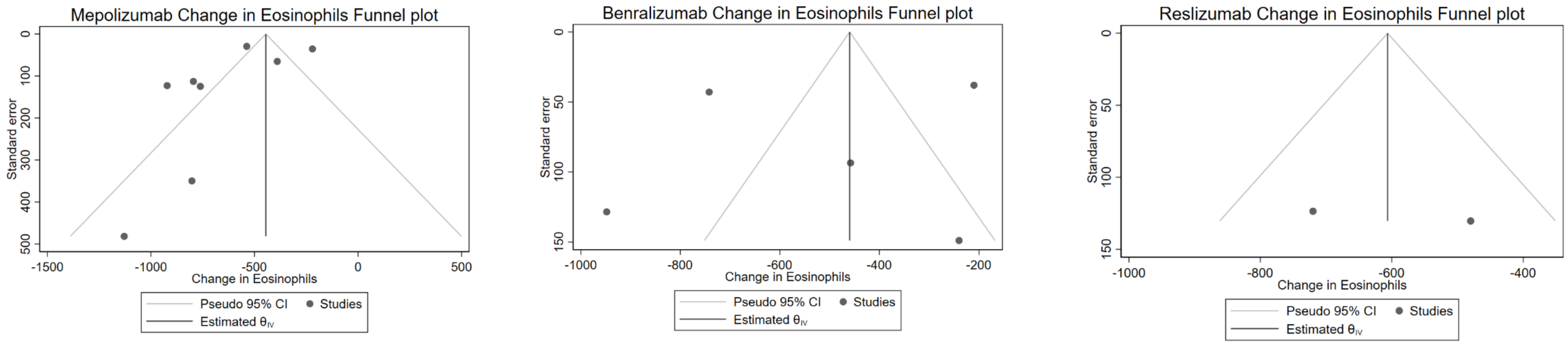

CI (Confidence Interval).

Supplementary Figure 22: Change in Steroids Funnel Plot

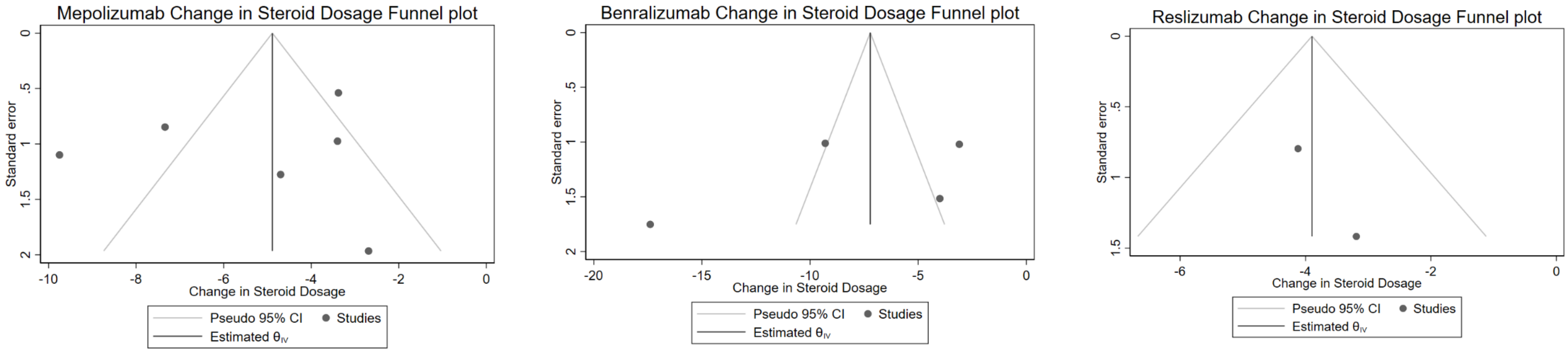

CI (Confidence Interval).
